# Cockayne syndrome mice reflect human kidney disease and are defective in de novo NAD biosynthesis

**DOI:** 10.1038/s41418-025-01522-7

**Published:** 2025-05-15

**Authors:** Komal Pekhale, Vinod Tiwari, Mansoor Hussain, Christy C. Bridges, Deborah L. Croteau, Moshe Levi, Avi Z. Rosenberg, Briana Santo, Xiaoping Yang, Tomasz Kulikowicz, Xiaoxin X. Wang, Jong-Hyuk Lee, Vilhelm A. Bohr

**Affiliations:** 1 DNA repair section, National Institute on Aging, National Institutes of Health, Baltimore, MD 21224 USA; 2 Cecil H. and Ida Green Center for Reproductive Biology Sciences, University of Texas Southwestern Medical Center, Dallas, TX 75390 USA; 3 Department of Biomedical Sciences, Mercer University School of Medicine, Savannah, GA 31404 USA; 4 Computational Biology & Genomics Core, Laboratory of Genetics and Genomics, National Institute on Aging, National Institutes of Health, Baltimore, MD 21224 USA; 5 Biochemistry and Molecular & Cellular Biology, Georgetown University Medical Center, Washington, DC USA; 6 Department of Pathology Johns Hopkins University School of Medicine, Baltimore, MD USA; 7 Johns Hopkins University School of Medicine, Baltimore, MD USA; 8 Center for Gerontology, Mercer University, Macon, GA 31207 USA; 9 Danish Center for Healthy Aging, University of Copenhagen, 2200 Copenhagen, Denmark

**Keywords:** Gene expression, Enzyme mechanisms

## Abstract

Cockayne Syndrome (CS) is a premature aging disorder caused by mutations in the CSA and CSB genes involved in DNA metabolism and other cellular processes. CS patients display many features including premature aging, neurodegeneration, and kidney abnormalities. Nicotinamide dinucleotide (NAD^+^) deprivation has been observed in CS patient-derived cells. NAD^+^ has essential roles in regulating cellular health, stress responses, and renal homeostasis. While kidney dysfunction is a common feature in CS patients, its molecular pathogenesis is not understood. Here, we report that severe kidney pathology is present in CS A and B mice. We find that the NAD^+^ biosynthetic pathways are impaired in kidneys from these mice. Using human renal tubular epithelial cells, we show that CSA/B downregulation causes persistent activation of the ATF3 transcription factor on the quinolinate phosphoribosyl transferase gene locus, a rate-limiting enzyme in de novo NAD^+^ biosynthesis in the kidney, causing impaired transcription and deficient NAD^+^ homeostasis.

## Introduction

Cockayne syndrome (CS) genes play important roles in DNA repair and transcription [1]. Mutation in these genes results in CS, a profoundly debilitating disorder characterized by short stature, microcephaly, premature aging, neurodegeneration, photosensitivity, vision impairment, hearing loss, and bone and kidney abnormalities [2]. There are two complementation groups, CSA and CSB which are mainly caused by defects in DNA repair genes ERCC8 and ERCC6, respectively. About 80% of the patients belong to group B, while the remainder are in group A. While CSA and CBS proteins have different functions, the clinical features of the patients are similar in the two complementation groups. The molecular basis of CS has traditionally been attributed to defects in transcription and transcription-coupled nucleotide excision repair (TC-NER). However, recent work suggests that defects in base excision DNA repair and mitochondrial functions also play important roles [3]. One of the key molecular characteristics of CS (patient-derived) cells is the reduced abundance of nicotinamide dinucleotide (NAD^+^) [4]. NAD^+^ is a critical cofactor for redox reactions in the cytosol and mitochondria, which enables energy metabolism of the cell. NAD^+^ is also needed for the activity of several enzymes involved in mitochondrial biogenesis, mitophagy, and energy metabolism. Damaged DNA recruits poly-ADP-ribosyl polymerase 1 (PARP1) and activates its poly-ADP-ribosylation activity in situ. As intracellular NAD^+^ is a substrate for ribosylation, persistent activation of PARP leads to the depletion of intracellular NAD^+^ and impairment of many downstream processes, including DNA repair and mitochondrial functions. PARPs transfer ADP-ribose from NAD^+^ to itself, histones, and other proteins at sites of DNA damage. CSA is recruited to stalled RNA pol II by CSB following UV DNA damage [5] and is involved in recruitment of the TC-NER machinery in response to UV damage via the DDB1-CUL4- E3 ubiquitin ligase complex [6, 7]. CSB protein displaces PARP1 from its binding sites and terminates its PARylating activity [8]. Thus, CSB deficiency leads to further depletion of intracellular NAD^+^ and lowers the activity of sirtuin proteins and other NAD^+^ dependent enzymes [9]. This has been observed in cells derived from CS patients and in model animal systems such as mice and worms [10].

NAD^+^ has critical roles in the generation of ATP from fuel substrates and acts as a substrate for important enzymes that regulate cellular health and stress responses. It mediates hydrogen transfer in oxidative and reductive metabolic reactions and is also a co-substrate for PARP, CD38/157, and class III NAD^+^-dependent deacetylases or sirtuins [11]. Sirtuins (i.e., SIRT1) play key roles in responding to nutritional and environmental perturbations such as fasting, calorie restriction, DNA damage, and oxidative stress. SIRT1 deacetylation of FOXO isoforms and p53 may reduce apoptotic signaling [12], while SIRT1 deacetylation of NF-κB attenuates inflammatory responses [13]. Activation of SIRT1 by caloric restriction or by a ketogenic diet delays the aging process. SIRT3 is present in the mitochondria and is important for many reactions involved in maintaining mitochondrial health. NAD^+^ plays a key role in maintaining healthy mitochondria through activation of SIRT1 [14] and SIRT3 [15], which regulate transcription-mediated mitochondrial biogenesis [14] and enhance mitophagy to remove damaged mitochondria [16].

Renal tubules are a major component of nephrons in the kidneys. They reabsorb and secrete fluid, electrolytes, and other substrates to maintain proper fluid and solute homeostasis. The tubules are highly metabolically active and require a constant supply of ATP to provide the energy required to pump solutes across unfavorable gradients. Proximal tubular epithelial cells possess abundant mitochondria to support their high energy demand. Thus, NAD^+^ deprivation leads to mitochondrial impairment, which in turn, triggers the energy metabolism alterations associated reactive oxygen species production to promote inflammation in these cells, resulting in renal injury [17].

Renal failure is an important feature in CS patients and was first reported in the mid-1960s [18]. One of the latest case reports investigating renal disease in CS found that approximately 70% of CS patients have renal complications [19]. Patients with CS experience a gradual loss of renal function, characterized by nephron loss, arteriolosclerosis, and glomerular hyalinosis, manifesting as hypertension, proteinuria, and hyperuricemia [19]. Despite the importance of NAD^+^ in renal homeostasis, the molecular pathogenesis of renal injury in CS patients is not understood, and the link between NAD^+^ and kidney dysfunction in CS patients has not been explored.

The present study was designed to test the hypothesis that the decreased intracellular NAD^+^ results in renal disease in CS patients. We found renal atrophy, decreased glomerular filtration, and various types of lesions centering around the tubular epithelial cells in the kidneys of CS mice. Using RNA-sequencing analyses, we detected impaired NAD^+^ biosynthetic pathways in CS kidneys. We used CSA/B knockdown in cultured human proximal tubular epithelial cells to model the tubular epithelial cells of CS kidneys and found that this downregulation causes cellular NAD^+^ deprivation. Moreover, CSA/B knockdown caused transcription repressor ATF3 activation on the quinolinate phosphoribosyl transferase (QPRT) gene locus, a rate-limiting enzyme in de novo NAD^+^ biosynthesis in the kidney, causing impaired transcription. With these data, we propose a novel model for renal impairment in CS patients and show that CSA/B is critical in maintaining renal NAD^+^ homeostasis by ATF3-mediated transcriptional regulation of QPRT.

## Materials and methods

### Animals

Mouse models of *CSA*^*−/−*^ (knockout mice), *CSB*^*m/m*^ (mice carrying a premature stop codon in exon 5 to mimic the K337 stop truncation mutation from a CS patient), and wild-type (WT) on a C57BL/6 J background in the age range of 45–68 weeks were used for all the experiments, unless otherwise specified.

### Antibodies

The antibodies used were as follows: CSA (Abcam, ab137033), CSB (Abcam, ab96089), QPRT (Biorybt, orb317756), ATF3 (Abcam, ab207434), β Actin (SantaCruz, sc47778), GAPDH (Cell Signallng Technology, 2118), Havcr-1 (ThermoFischer scientific, PA5-98302).

### Cell Culture

HK-2 (Human kidney proximal tubular epithelial cell line (ATCC CRL-2190)) cells were cultured in Keratinocyte serum-free medium (K-SFM) containing bovine pituitary extract (BPE), human recombinant epidermal growth factor (EGF) in a humidified chamber under 5% CO_2_ at 37 °C.

### siRNA knockdown

siRNAs for CSA (SR319723A; SR319723C) and CSB (SR320072A; SR320072B) and non-targeted siRNA (SR3005) were purchased from Origene (Rockville, MD, USA). siRNA was diluted with K-SFM to a final concentration of 50 nM, mixed with JetPrime (Polyplus transfection, Illkirch-graffenstaden, France), incubated for 15 min at room temperature, and transfected into target cells according to the manufacturer’s instructions. Twenty‐four hours after transfection, the media was replaced. Cells were harvested 72 h after transfection and used for the experiments.

### NR supplementation

72 h post siRNA transfection, HK-2 cells were supplemented with 1 mM NR (Chromadex, CA, USA) for 24 h. Following NR treatment, the cells were harvested and used for further experiments.

### Immunoblotting

Cells were harvested, washed with PBS, and lysed in 1X RIPA buffer (ThermoFisher scientific, Waltham, MA, USA) with protease inhibitor cocktail (Cell Signaling Technology, Danvers, MA, USA). Protein concentrations were determined by a BCA (ThermoFisher scientific) assay. Equal concentrations of proteins were loaded on Mini-PROTEAN® precast gels (Bio-Rad, Hercules, CA, USA) for SDS-PAGE. Proteins were transferred on a PVDF membrane (0.45 μM pore size) using a Mini Trans-Blot® Cell transfer system (Bio-Rad). After blocking the membrane in 3% nonfat milk in TBS-T (Tris buffered saline with 0.1% Tween-20), it was incubated overnight with primary antibodies at 4°C, followed by anti-rabbit HRP-conjugate IgG or anti-mouse HRP-conjugate IgG. Immunoblot signals were detected using an ECL detection kit (ThermoFisher scientific). Immunoblots were imaged and quantitated using a Bio-Rad Chemidoc platform.

### NAD^+^ quantification

Kidneys were dissected in cold PBS and placed in NADH/NAD Extraction Buffer (ab65348, abcam, Boston, MA, USA). Following homogenization with a micro pestle, an NAD/NADH Assay Kit (ab65348, abcam) was used to quantify NAD^+^ and NADH levels.

HK-2 cells were harvested after siRNA transfection, washed with PBS, and lysed in NADH/NAD Extraction Buffer (ab65348, abcam). NAD^+^ and NADH levels were quantified according to the manufacturer’s instructions. Data were normalized to scrambled siRNA treated (SCR) HK-2 cells.

### Quantitative real-time PCR (qPCR)

Total RNA was extracted from cells using a Nucleospin Triprep kit (Macherey-Nagel, Düren, Nordrhein-Westfalen, Germany) or Purelink RNA isolation kit (Invitrogen, CA, USA). One microgram of total RNA was reverse transcribed using the iScript™ cDNA Synthesis Kit (Bio-Rad). qPCR was performed using the DyNAmo HS SYBR Green qPCR Kit (F-410L, ThermoFisher scientific) with the CFX Connect Real-time PCR Detection System (Bio-Rad). Ct values were normalized to values for *GAPDH* for HK-2 cells while *Actin* was used for mice. Primer sequences used are listed in Supplementary Table 1. Data are reported as mean ± standard deviation.

### RNA sequencing

RNA from the mice were isolated using RNeasy (Macherey-Nagel). Library construction and sequencing were performed by Novogene (Sacramento, CA, USA). The samples were run on the NovaSeq 6000. The samples have >95% bases with Q20 and above. Samples were aligned to the reference mouse genome (mm10) and junctions using HISAT2 (v2.0.5) software. Differential expression analysis of the groups (two biological replicates per condition) were performed using the DESeq2 R package (1.20.0). The resulting P-values were adjusted using the Benjamini and Hochberg’s approach for controlling the false discovery rate (FDR). Genes with an adjusted *P* value ≤ 0.05 found by DESeq2 were assigned as differentially expressed with foldchange of 2 and 0.5.

Gene Ontology (GO) enrichment analysis of differentially expressed genes was implemented by the ClusterProfiler R package, in which gene length bias was corrected. GO terms with corrected *P* value less than 0.05 were considered significantly enriched by differential expressed genes. ClusterProfiler R package was used to test the statistical enrichment of differential expression genes in KEGG pathways.

GEO accession number is GEO # GSE246444.

### Biological assessment

Whole blood samples from the mice were collected at sacrifice by cardiac puncture. Blood was allowed to stand for 30 min and centrifuged at 2000 × *g* for 15 min. Serum was transferred to a new tube and different biochemical parameters were checked. Urine was collected from the mice by holding the mice over the collection tube, until sufficient amounts were collected for biochemical analysis. Biochemical analysis for Serum and urine was done by IDEXX Laboratories, Inc (Westbrooke, Maine, USA). Urine chemistry values were normalized to each animal’s respective urine creatinine level.

### Detection of kidney toxicity markers

Serum and urine collected from the mice were analyzed using a FirePlex® kidney toxicity immunoassay (Abcam, ab235661), to detect the levels of clusterin, cystatin C, havcr1, lipocalin-2, and osteopontin as per the manufacturer’s protocol.

### Chromatin immunoprecipitation (ChIP)

ChIP was performed as described [20], with slight modifications. Briefly, HK-2 cells were incubated in 1% formaldehyde PBS for 10 min at room temperature. The solution was brought to a final concentration of 0.125 M glycine, and cells were harvested by centrifugation, washed three times in cold PBS, then incubated in buffer A (5 mM PIPES (pH 8.0), 85 mM KCl, 0.5% NP-40, and protease inhibitor cocktail (GenDEPOT, Katy, TX, USA)). Cell extracts were centrifuged, and pelleted material was resuspended in buffer B (100 mM Tris-Cl (pH 8.1), 1% sodium dodecyl sulfate (SDS), 10 mM EDTA, and protease inhibitor cocktail). Chromatin was sheared using an S-450 sonicator (Branson, Danbury, CT, USA.). An aliquot containing 500 μg DNA was diluted 10-fold in IP buffer (0.01% SDS, 1.1% Triton X-100, 1.2 mM EDTA, 16.7 mM Tris-Cl (pH 8.1), 167 mM NaCl, and protease inhibitor cocktail) and incubated with primary antibody for ATF3 (abcam ab207434) overnight at 4°C. Samples were incubated 30 min at 4 °C with protein A/G-linked magnetic beads. Beads were washed sequentially with TSE150 (1% Triton X-100,0.1% SDS, 2 mM EDTA, 20 mM Tris-Cl (pH 8.1), and 150 mM NaCl), TSE500 (1% Triton X-100, 0.1% SDS, 2 mM EDTA, 20 mM Tris-Cl (pH 8.1), and 500 mM NaCl), and Buffer III (0.25 M LiCl, 1% NP-40, 1% sodium deoxycholate, 1 mM EDTA, and 10 mM Tris-Cl (pH 8.1)) and then washed twice with TE (pH 8.0) for 10 min. Chromatin was eluted with elution buffer (1% SDS and 0.1 M NaHCO3 (pH 8.0)) and incubated overnight at 65°C in 200 mM NaCl to reverse cross-linking. Aliquots (500 μL) were incubated at 50°C after addition of 10 μL 0.5 M EDTA, 20 μL 1 M Tris (pH 6.5) and 4 μL Proteinase K (20 mg mL^−1^), extracted sequentially with phenol/chloroform/isoamyl alcohol. Nucleic acids were pelleted by centrifugation for 30 min at 4°C after addition of 1 μL of 20 mg mL^−1^ glycogen, 20 μL of 5 M NaCl and 500 μL of isopropanol. Pellets were washed with 70% ethanol, dried and resuspended in nuclease-free water.

### qPCR of ChIP products

ChIP products were subject to qPCR using the DyNAmo HS SYBR Green qPCR Kit (F-410L, ThermoFisher scientific) with the CFX Connect Real-time PCR Detection System (Bio-Rad). Experimental values were normalized to the values of 1% input chromatin. Concentrations were estimated using the 2^−ΔΔCT^ calculation method. The sequences of the primers are listed in Supplementary Table 1.

### Histological analysis

Mice were anesthetized deeply with isoflurane and then perfused with 1X phosphate buffered saline (PBS) through the heart. Kidneys were removed, fixed in 4% paraformaldehyde (PFA) for 24 h at 4 °C, and then washed with PBS. Tissue preparation, mounting, and staining was done by Histoserv (Germantown, MD, USA). Kidneys were trimmed, processed, and embedded in paraffin blocks. Formalin-fixed, paraffin-embedded (FFPE) blocks were cut into 5 µm thick sections and placed on Superfrost slides. To perform immunohistochemistry, slides were deparaffinized using xylene and rehydrated using graded concentrations of ethanol and water. Heat-mediated antigen retrieval was then performed using a citrate-based buffer. Slides were then incubated with the primary antibody, either QPRT (orb317756, Biorybt, Cambridge, UK) (1:1000) or Havcr-1 (PA5-98302, ThermoFisher scientific) (1:1600) followed by a goat-anti-rabbit-HRP-conjugated secondary. Detection was then developed with DAB and counterstained with hematoxylin. Slides were subsequently dehydrated, cleared, mounted with a permanent mounting medium, and scanned using a Leica AT2 system at 0.25 µm/px. Image analysis and quantification for Havcr1 was using Qupath [21], while QPRT images were analyzed and quantitated by ImageJ [22].

Sections (4 μm thick) cut from 10% formalin-fixed, paraffin-embedded kidney samples were used for periodic acid-Schiff (PAS) staining or picrosirius red (PSR) staining from Histoserv. Quantifications were performed in a masked manner. Using coronal sections of the kidney, 30 consecutive glomeruli per mouse, with 3–5 mice per group, were examined for evaluation of glomerular mesangial expansion. The index of mesangial expansion was scored based on the ratio of mesangial area to glomerular tuft area. The mesangial area was determined by assessment of PAS-positive and nucleus-free areas in the mesangium using the ScanScope image analyzer (Aperio Technologies, Vista, CA). Using color deconvolution algorithm on ScanScope image analyzer, the quantification of PSR staining was expressed as a percentage of the red positive area on the total cortical surface.

### TUNEL assay

A TUNEL assay was performed using the Click-iT™ Plus TUNEL Assay for In Situ Apoptosis Detection kit (Invitrogen, CA, USA). In brief, HK-2 cells were transfected with CSA/B siRNAs. After 72 h of transfection, cells were supplemented with/without 1 mM NR for 24 h. Following NR treatment, cells were washed three times with PBS, fixed with 4% formaldehyde for 30 min, and permeabilized with 0.5% Triton X-100 for 10 min. Then, the TUNEL probe was incubated with the cells for 4 h at room temperature. After staining with Hoechst to label the nucleus for 3 min at room temperature, the samples were observed and analyzed under an inverted microscope (Zeiss, Observer Z.1).

TUNEL staining for kidney sections was performed using a TUNEL assay kit from abcam (ab20638), following the manufacturer’s protocol.

### p57 immunohistochemistry

Paraffin-embedded kidney section slides were deparaffinized and rehydrated using graded concentrations of ethanol and water. Heat-mediated antigen retrieval was then performed using an EDTA-based buffer. After retrieval, slides were then incubated with the p57 primary antibody (ab75947, abcam, Waltham, MA) (1:100) in antibody diluent (BSB 0114, BioSB Inc., Santa Barbara, CA) followed by a goat-anti-rabbit-HRP-conjugated secondary antibody (AF835, R&D system, Minneapolis, MN). Detection was then developed with an AEC substrate (SK-4205, Vector Laboratories, Newark, CA) and counterstained with hematoxylin. Slides were mounted with Vecta Mount AQ mounting medium (Vector Lab, H-5501) and scanned using a MoticEasyscan scanner (Motic, Vancouver, Canada). The slides were stripped and re-stained with the periodic acid Schiff stain reagents (87007, Richard-Allan Scientific, Kalamazoo, MI). Images were re-collected by scanning p57, and periodic acid-Schiff-stained images for each slide, which were then fused into a single image by HALO software (Indica Labs, Albuquerque, NM). The average number of podocytes per glomerulus was calculated by manually counting 50 glomeruli for each case section.

### Glomerulus quantification from digital histology

Glomeruli were manually annotated using the Aperio ImageScope digital histology viewer [v12.3.3, Leica Biosystems]. All subsequent computational image analysis was completed in MATLAB [vR2024b]. Whole-slide glomerulus masks were computed from xml annotation files, and glomerulus areas were computed using the regionprops function. Mesangium was segmented from each glomerulus using color deconvolution with HPAS stain vectors extracted using the Fiji ImageJ Color Deconvolution plug-in. The percentage mesangial area was computed as the sum of PAS+ pixels indexed to glomerular area.

### Glomerular area feature mapping

The areas of glomeruli from all wild-type kidneys were pooled, and a histogram was constructed using a sequential color heatmap. In the color heatmap, low glomerulus areas were represented by dark red, while the highest glomerulus areas were represented by dark blue. The glomeruli from all KO kidneys were then assigned to the corresponding histogram bin based on their glomerular area. Glomeruli in whole-slide image segmentation masks were re-colored according to the histogram bin that they were assigned, producing images where glomeruli are colored based on area relative to the global distribution of glomerulus areas across the dataset. Re-colored glomerulus segmentation masks were superimposed on whole-slide H&E images to visualize the underlying glomerular histologic presentation. All image processing was completed in MATLAB.

### Tubule quantification from digital histology

ARTSA, a desktop application for renal tubule quantification from whole-slide histopathology, was used to automatically segment and quantify tubules and tubule nuclei [23, 24]. Morphological features extracted from renal tubules included area, perimeter, eccentricity, solidity, and percentage tubule (total tubule area per kidney indexed to total kidney area). Percentage nuclear area per tubule was computed as the total nuclear area per tubule indexed to tubule area. Textural features extracted from tubule nuclei included first and second-order pixel statistics. First order nuclear pixel statistics included minimum, maximum, range, mean, variance, standard deviation, median, interquartile range, 10th percentile, 90th percentile, mean absolute deviation, root mean absolute deviation, root mean squared, skewness, kurtosis, uniformity, and entropy. Second order statistics included gray-level co-occurrence matrix (GLCM) contrast, correlation, energy, and homogeneity.

### Statistical analysis

GraphPad Prism (GraphPad Software, Inc.) was used to perform the statistical analysis. A two-tailed t test was used to compare individual group. A two-way ANOVA was used to compare multiple samples within a single group.

For all computed histological image features, normality was assessed with the Anderson Darling test, and variance was assessed with Bartlett’s (parametric) or Levene’s (non-parametric) test. For comparisons among groups, ANOVA (parametric) or Kruskal-Wallis (non-parametric) tests were completed, with corresponding post-hoc tests (Tukey’s and Dunn’s tests, respectively).

## Results

### Cockayne syndrome mouse models show kidney abnormalities

**Fig. 1:**
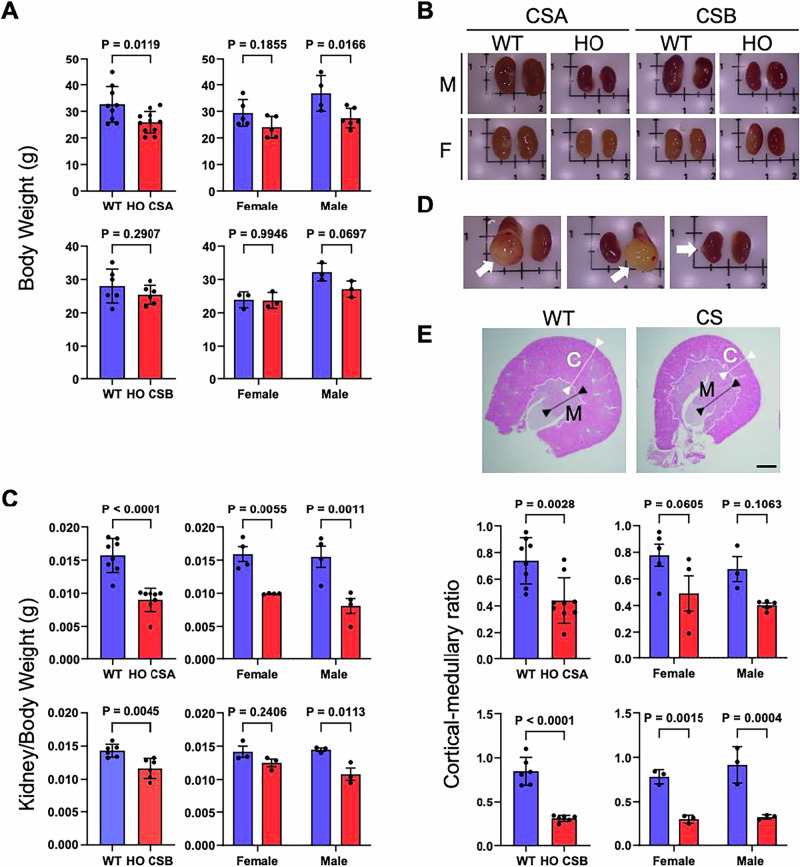
Cockayne syndrome (CS) mice models exhibits kidney abnormalities. **A** Body weight measurement of CS mice compared to wild type. For CSA (aged 47–68 weeks), 5 females and 4 males were used as controls (WT) and 5 females, and 6 males were used as homozygous knockout experimental group (HO CSA). For CSB (aged 45–68 weeks), 3 males and females were used for both control (WT), and the experimental groups (HO CSB). **B** Kidneys from each group were isolated and weights were normalized to the total body weight. Scale bar, 5 mm. **C** Representative image of CS mice kidneys, and (**D**) with arrows indicating fluid filled-blisters on the surface. **E** Transverse section of WT and CS kidneys (aged 45–68 weeks). Lengths of the renal cortex (C) and medulla (M) were measured and cortical to medullary ratio were compared. Scale bar, 1 mm. Data are presented as mean ± SD (*n* ≥ 3). A two‐way ANOVA Sidak test was used for the comparisons between males and females. p-values indicated (Blue bar, WT; Red bar, HO CSA/CSB).

Our lab utilizes mouse models of *CSA*^*−/−*^ (knockout mice) [25] and *CSB*^*m/m*^ (mouse carrying a premature stop codon in exon 5, mimicking the K337 stop truncation mutation in a CS patients) [26], which mimics human CS and range of CS features. In previous reports, CSA-deficient mice have been found to be grossly indistinguishable from heterozygous and wild-type litter mates up to the age of 24 months [27]. In contrast, CSB-deficient mice are small with a highly significant reduction in body weights in males. Reduced body weights are less pronounced in female mice [25]. 47-68 weeks old male and female CSA mice showed body weight loss, especially in males. 45-68 weeks old CSB mice did not show body weight differences compared to controls in females; however, males showed a trend toward weight loss (*p* value 0.07) (Fig. 1A). In both CSA and CBS mice, we found decreased weight and volume of the kidneys compared to WT mice (Fig. 1B, C). Upon visual inspection, the CS kidneys were noticeably pale compared to WT (Fig. 1B and Supplementary Fig. 1), indicating a possible vasculature reduction or vascular stricture due to fibrosis, which could impact the glomerular filtration rate (GFR). We found fluid filled-blisters on the external surface of the renal cortex in many of the CS kidneys, (Fig. 1D). Moreover, the cortical to medullary ratio of kidneys was reduced by approximately 50% in CSA and CSB animals compared with WT (Fig. 1E). The renal cortex contains the glomeruli that are responsible for filtration and the medulla consists of the medullary collecting ducts, loops of Henle, vasa recta, and the interstitium, which are essential for the regulation of urine concentration. Decreased cortical mass in CS mice suggests that these animals may have insufficient glomerular filtration due to decreased number/size of glomeruli, compared to WT. This may also correlate to the paleness observed in CS kidneys (Fig. 1B and Supplementary Fig.1 A), as the cortex contains numerous capillaries.

#### Histopathological features of CS mice kidneys

**Fig. 2:**
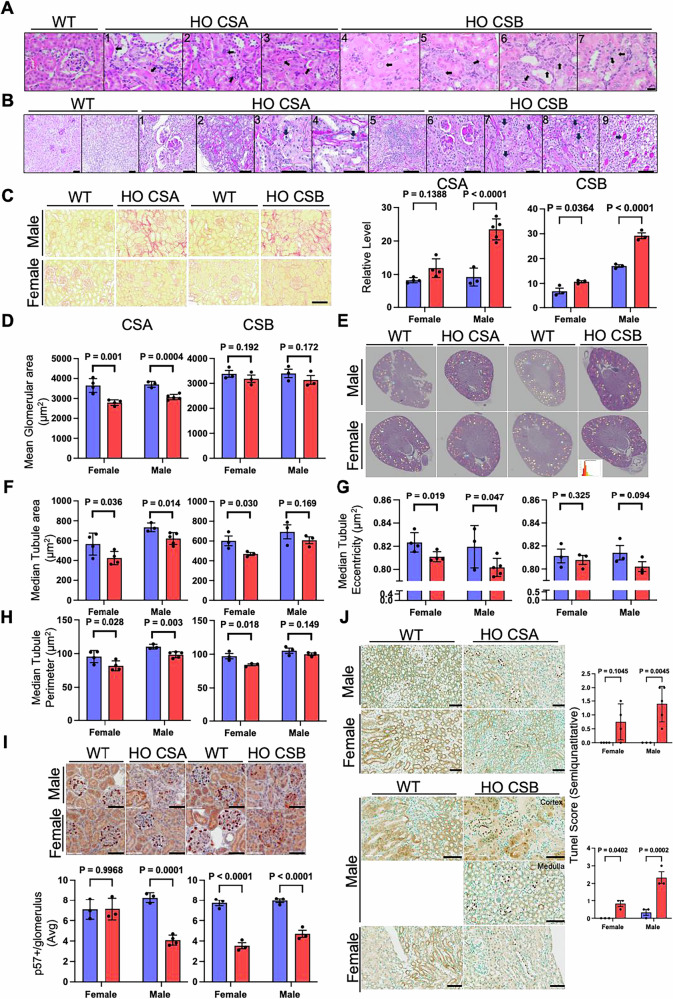
Histopathological and Morphometric analysis of CS mice. **A** Histology findings of different lesions in male CS mice compared to wild type (aged 17–21 weeks) and examples include (1) Cytomegaly; (2) Karyomegaly; (3) Hyaline casts; (4) Karyomegaly; (5) Tubular regeneration; (6) Tubular regeneration, Karyomegaly, and epithelial degeneration; (7) Karyomegaly and epithelial degeneration. Scale bar, 20 μm. **B** Histology findings of different lesions in CSA and CSB mice compared to wild type (aged 45–68 weeks) and examples indicated include (1) hyaline glomerulopathy; (2) cortical scarring; (3) tubular nuclear enlargement consistent with tubular injury and occasional apoptotic cells; (4) mitotic figures; (5) foci of plasma cell rich inflammation; (6) hyaline glomerulopathy; (7) tubular cytoplasmic vacuolation; (8) focal tubular epithelial karyomegaly; (9) bland hyaline casts. Scale bar, 60 µm. **C** Representative image (left) and quantification (right) of PSR staining in CS mice. Scale bar, 100 µm. **D** Mean glomerular area quantification. **E** Whole-slide H&E images with glomeruli color-coded according to their corresponding histogram bin by digital histology (refer to “Materials and Methods” for details). Tubular feature quantifications (**F**) Median Tubular area, (**G**) Median tubule eccentricity, and (**H**) Median Tubule Perimeter by digital histology. **I** p57 immunohistochemistry (IHC) in kidney sections of CSA and CSB mice. Representative IHC image (Top) and quantification (bottom) of p57 staining in CS mice is shown. Scale bar, 60 µm. **J** Representative and quantification of TUNEL staining in CS mice kidney sections. Scale bar, 60 µm. Data are presented as mean ± SD (*n* ≥ 3). A two‐way ANOVA Sidak test was used for the comparisons between males and females. *p* values indicated (Blue bar, WT; Red bar, HO CSA/CSB).

Pathologist blinded morphological analyses of 17–21 week-old male kidneys stained with hematoxylin & eosin (H&E) show details of the pathology in the CS (CSA^−/−^ and CSB^m/m^) kidneys (Fig. 2A). Renal tubular epithelial cells appear swollen with cytomegaly and karyomegaly. Karyomegaly (nuclear size enlargement) and cytomegaly (increase in cell size) are characterized by hyper-chromatic (abnormal intensity of color, indication of an elevated level of chromatin) nuclei with multiple nucleoli and irregularly shaped proximal convoluted tubular cells, resulting from nucleic acid replication without nuclear division. These are generally considered as preneoplastic, leading to tubule neoplasia in chronic disease studies [28]. Also, we detected necrotic cells with enlarged nuclei and clear cytoplasm. A small number of degenerating tubules were characterized by increased basophilia (increased nuclear to cytoplasmic ratio), small cells, and thickened basement membranes. Potential tubular injury indicated by hyaline casts and subsequent regeneration most likely led to reduced glomerular filtration to prevent dilation and further injury of the necrotic tubule. Regenerative cells spread over the basement membrane where the necrotic epithelium has been damaged [29]. We found that these defects in CS kidneys are localized primarily in tubular epithelial cells (Fig. 2A). Moreover, the lesions are morphologically similar to features of chronic progressive nephropathy (CPN), which is an age-related renal disease affecting rodents, with unknown etiology. Features of this disease include basophilic tubules with conspicuously thickened basement membranes and crowded nuclei [30]. This finding is consistent with findings in CS patients with thickening of the glomerular basement membrane, mesangial expansion and tubule atrophy [31].

Upon deeper histopathologic analysis of older mice (45–68 weeks), more advanced lesions were observed (Fig. 2B). Hyaline glomerulopathy, a low-prevalence lesion in both CSA and CSB KO mice compared to WT controls, was more frequent in CSB than in CSA. This lesion, consistent with segmental sclerosis, is typically associated with aging in mice.

Tubulointerstitial lesions varied in presentation. Both CSA and CSB mice exhibited areas of cortical scarring with tubular atrophy. Additionally, focal reactive nuclear changes were noted in medullary and cortical tubules, accompanied by bland PAS-positive casts, scattered apoptotic bodies, and mitotic figures.

To assess mesangial expansion, we performed periodic acid-Schiff (PAS) staining, but no significant changes were found in the CS kidneys (Supplementary Fig. 1B). In contrast, picrosirius red (PSR) staining revealed significantly increased fibrosis, particularly in male CS kidneys (Fig. 2C and Supplementary Fig. 2A), while female CS kidneys showed a relatively mild increase. Notably, CSA mice exhibited a significantly reduced mean glomerular area (Fig. 2D, Supplementary Fig. 2B). A similar trend was observed in CSB mice, though it did not reach statistical significance. To visualize the glomerular changes in CS animals, glomerular areas from all wild-type kidneys were combined to generate a histogram using a sequential color heatmap (Fig. 2E, histogram image within the female HO CSB). In this heatmap, smaller glomerular areas were depicted in dark red, while the largest areas appeared in dark blue. In both male and female WT CSA and CSB mice, glomerular areas were generally larger, as indicated by a higher prevalence of orange and yellow glomeruli, whereas KO mice predominantly exhibited red-shaded glomeruli, signifying smaller glomerular areas across the tissue (Fig. 2E and Supplementary Fig. 2C). Tubular morphometry analysis further revealed a significantly decreased tubular area in all groups except for CSB males (Fig. 2F and Supplementary Fig. 2D). A significant reduction in tubular eccentricity was observed exclusively in CSA mice (Fig. 2G and Supplementary Fig. 2E), while tubular perimeter was significantly decreased in all groups except for CSB males (Fig. 2H and Supplementary Fig. 2F). These findings suggest potential tubular atrophy.

To further investigate glomerular pathology, we performed p57 immunohistochemical staining and found that the podocyte count per glomerulus was significantly reduced in all groups except for CSA females (Fig. 2I).

Since NAD^+^ dysregulation can drive cell death, including apoptosis [32], we assessed its potential contribution to CSA and CSB pathology using TUNEL staining. An increased number of apoptotic cells was observed in the distal nephron, with greater TUNEL positivity in males compared to females (Fig. 2J). Furthermore, nephron involvement was more extensive in CSB than in CSA. In CSA, apoptosis was largely restricted to medullary tubular segments, whereas in CSB, it extended to cortical segments of the distal nephron as well.

Overall, the pattern of renal parenchymal injury is consistent with tubulocentric injury, with secondary evolving glomerular damage. No notable pathological findings were observed in WT mice, confirming that the observed pathology is genotype-specific.

#### Impairment of kidney functional markers in CS mice

**Fig. 3:**
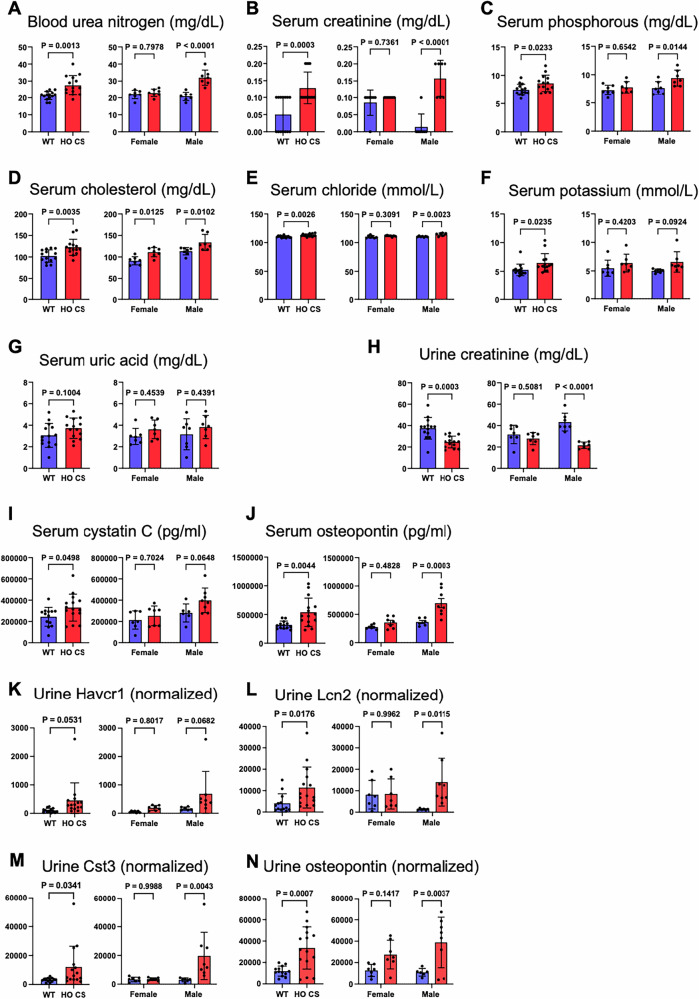
Kidney dysfunction in CS models. **A**–**G** Serum isolated from the whole blood samples and (**H**) urines collected from both male and female mice (aged 45–68 weeks) for both genotypes: WT and CS (CSA/B) mice and analyzed for the parameters indicated. **I**–**N** Kidney toxicity markers in serum and urine were determined by an immunoassay panel (refer to “Method” section for details). Data are presented as mean ± SD (*n* ≥ 3), An unpaired *t* test was used for comparing CS with WT. A two‐way ANOVA Sidak test was used for the comparisons between males and females. *p* values indicated (Blue bar, WT; Red bar, HO CSA/CSB).

Next, we performed serum chemistry analyses to further specify the renal impairment in CS mice. Blood urea nitrogen (BUN) (Fig. 3A) and creatinine (Fig. 3B), which are indicators of impaired kidney function, were increased significantly in CS mice. We also detected increased phosphorous and cholesterol levels in CS mice compared to WT (Fig. 3C, D). Moreover, we detected subtle, but significantly increased serum chloride and potassium levels (Fig. 3E, F). These changes were more pronounced in males, which is consistent with a published study showing that male patients with chronic kidney disease (CKD) have a greater decline in GFR than females [33]. However, while trends in females were observed, they did not reach statistical significance. Changes in serum uric acid level were not statistically significant but showed an increasing trend in CS mice (Fig. 3G). We did not observe any noticeable changes in other serum parameters (Supplementary Fig. 3). We found significantly decreased urine creatinine levels in CS mice (Fig. 3H), and creatinine normalized urine chemistry values showed almost two-fold increased urine calcium level in CS mice (Table 1). Most of the other values, such as sodium, potassium, phosphorous, urea nitrogen, chloride, and protein, were unchanged (Table 1 and Supplementary Fig. 3). Renal damage in CS mice was confirmed by elevated renal toxicity markers such as serum cystatin C (Cst3) and osteopontin (Spp1), as well as urine Spp1, lipocalin 2 (Lcn2) and Cst3 (Fig. 3I–N). In CS patients, increased serum creatinine and Cst3 correlate to decreased GFR [34]. Moreover, an inverse correlation between serum Spp1 and renal function was observed in CKD [35]. Again, these changes were more significant in males than in female CS mice. In addition to Cst3 and Spp1, other kidney injury markers hepatitis A virus cellular receptor 1 (Havcr1) and Lcn2 were also increased in serum and urine (Supplementary Fig. 4A, B) while urinary clusterin (Clu) was decreased (Supplementary Fig. 4B). Clu deficiency is suspected to cause glomerulopathy in aged mice [36]. This deficiency has also been shown in mice transitioning from acute kidney injury (AKI) to CKD, while upregulation of Havcr1 and Lcn2 act as early biomarkers for AKI [37]. Altogether, these data for the first time demonstrate the presence of severe renal damage in CS mice.

**Table 1:** Creatinine normalized urine chemistry data for WT and CS mice.

Parameter	CS WT	CS HO	CS WT-F	CS HO-F	CS WT-M	CS HO-M	CSA WT	CSA HO	CSA WT-F	CSA HO-F	CSA WT-M	CSA HO-M	CSB-WT	CSB-HO	CSB WT-F	CSB HO-F	CSB WT-M	CSB HO-M
Urine Sodium	2.34 ± 0.84	2.79 ± 0.88	2.33 ± 1.14	2.19 ± 0.41	2.35 ± 0.47	3.39 ± 0.82*	2.29 ± 0.57	2.79 ± 0.72	1.98 ± 0.57	2.38 ± 0.44	2.6 ± 0.41	3.2 ± 0.75	2.4 ± 1.17	2.79 ± 1.14	2.79 ± 1.69	1.94 ± 0.19	2.01 ± 0.33	3.64 ± 1.01
Urine Potassium	3.99 ± 0.9	4.02 ± 1.37	3.87 ± 1.08	3.12 ± 0.65	4.11 ± 0.74	4.92 ± 1.33	4.01 ± 0.76	4 ± 1.54	3.55 ± 0.37	3.19 ± 0.87	4.46 ± 0.8	4.82 ± 1.73	3.97 ± 1.14	4.04 ± 1.26	4.31 ± 1.67	3.02 ± 0.34	3.64 ± 0.3	5.06 ± 0.85
Urine Calcium	0.23 ± 0.07	0.37 ± 0.11***	0.26 ± 0.09	0.31 ± 0.09	0.21 ± 0.02	0.44 ± 0.11***	0.22 ± 0.07	0.4 ± 0.11**	0.22 ± 0.11	0.35 ± 0.06	0.21 ± 0.03	0.46 ± 0.14**	0.25 ± 0.06	0.33 ± 0.11	0.3 ± 0.03	0.26 ± 0.11	0.2 ± 0.01	0.4 ± 0.06*
Urine Phosphorus	4.52 ± 1.08	4.22 ± 1.84	4.23 ± 1.16	3.23 ± 0.76	4.8 ± 0.99	5.2 ± 2.12	5.15 ± 0.96	4.77 ± 2.2	4.77 ± 1.25	3.62 ± 0.77	5.53 ± 0.45	5.92 ± 2.67	3.67 ± 0.46	3.47 ± 0.95	3.5 ± 0.53	2.71 ± 0.32	3.83 ± 0.41	4.24 ± 0.63
Urine Urea Nitrogen	64.96 ± 10.38	62.82 ± 8.7	66.03 ± 10.13	57.78 ± 3.65	63.88 ± 11.32	67.85 ± 9.57	67.26 ± 7.21	61.44 ± 9.37	62.35 ± 5.83	57.22 ± 3.01	72.18 ± 4.77	65.67 ± 12.18	61.88 ± 13.7	64.64 ± 8.17	70.95 ± 13.92	58.53 ± 5	52.81 ± 5.33	70.75 ± 5.46
Urine Chloride	3.04 ± 1.32	3.64 ± 1.36	3.12 ± 1.66	2.77 ± 0.6	2.96 ± 1.02	4.51 ± 1.36	2.98 ± 0.81	3.73 ± 1.17	2.35 ± 0.35	3.09 ± 0.62	3.61 ± 0.57	4.36 ± 1.32	3.12 ± 1.91	3.52 ± 1.68	4.15 ± 2.3	2.33 ± 0.17	2.09 ± 0.79	4.7 ± 1.69
Urine Protein	10.24 ± 7.52	7.62 ± 6.48	3.38 ± 1.86	2.28 ± 0.8	17.1 ± 3.05	12.96 ± 4.86*	10.61 ± 8.44	5.79 ± 4.67	2.75 ± 0.94	1.79 ± 0.41	18.47 ± 0.7	9.78 ± 2.85***	9.74 ± 6.84	10.07 ± 8.12	4.22 ± 2.69	2.92 ± 0.75	15.27 ± 4.29	17.21 ± 3.37

Data are means ± SDM (*N* = 4, CSA WT/HO-F/M; 3, CSB WT/HO-F/M).

* *p* < 0.05 versus corresponding WT.

** *p* < 0.005 versus corresponding WT.

***p < 0.001 versus corresponding WT.

#### CS mice show altered Tryptophan metabolism

**Fig. 4:**
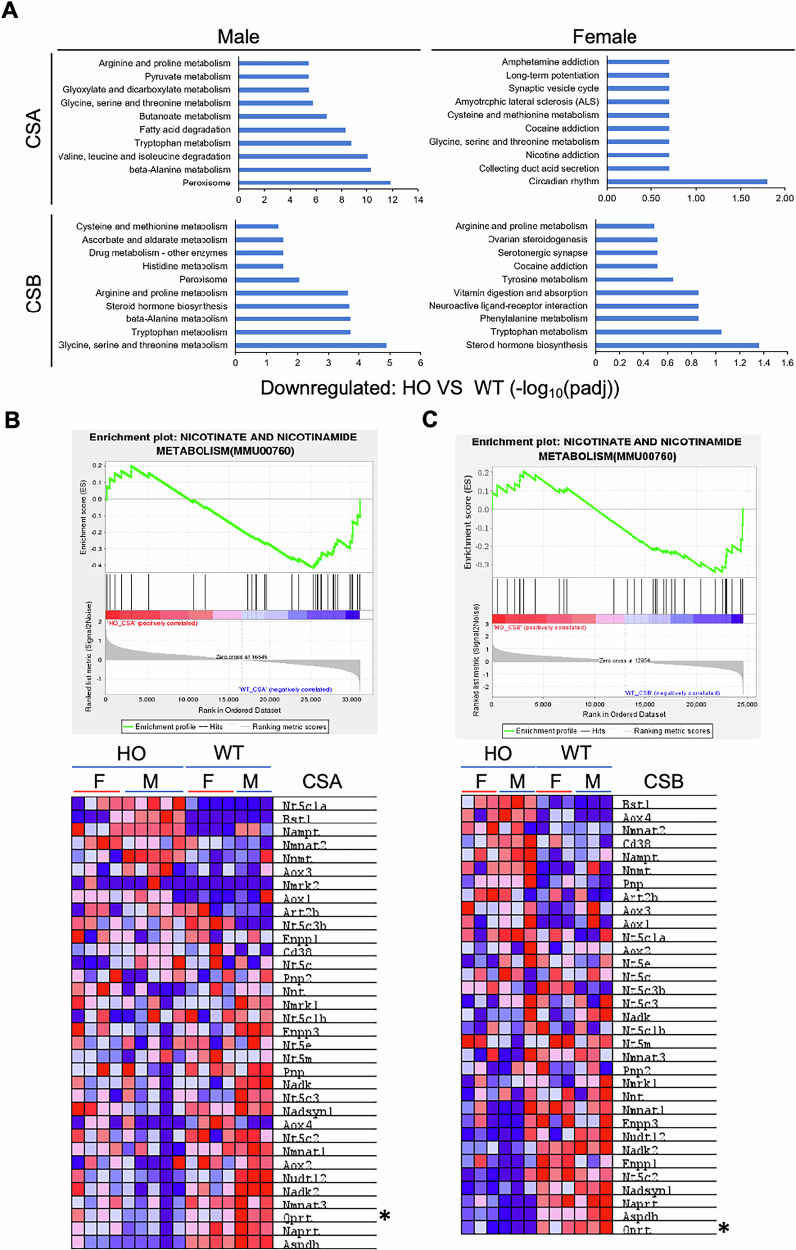
Impaired tryptophan metabolism in CS kidneys. Kidneys from CSA (4 females and 3 males for controls, 4 females and 5 males for HO CSA) and CSB (3 of each females and males for both controls and HO CSB) mice sets were isolated and RNA was purified. After RNA sequencing, differentially expressed gene sets (DEGs) were processed. **A** Significantly downregulated gene ontology (GO) set (*p* ≤ 0.05) analysis from DEGs. Gene set enrichment analysis (GSEA) plot and heatmap for the ‘Nicotinate and Nicotinamide metabolism’ panel from DEGs of CSA (**B**) and CSB (**C**) mice kidneys.

In order to identify potential mechanisms contributing to the kidney pathology, RNA-sequencing analysis (RNA-seq) of CS mouse kidney tissues was conducted. KEGG enrichment analysis showed that the expression changes in the set of genes involved in the tryptophan metabolism pathway were the most prominently and commonly downregulated in both CSA and CSB mice (Fig. 4A). As our observations of kidney features were most pronounced in males, we stratified these results by sex. Again, we detected more dramatic changes in male than female mice (Fig. 4A). Tryptophan metabolism via the kynurenine pathway is involved in conversion of quinolinic acid to NAD^+^, which is utilized by cells in several organs, including liver, kidney and spleen, for energy metabolism [38]. In the kidney, NAD^+^ availability relies heavily on the de novo biosynthetic pathway from tryptophan [39], and our finding suggests that CS kidneys may have impaired NAD^+^ homeostasis because of the altered NAD^+^ biosynthetic pathway. Impairment of this NAD^+^ synthesis pathway has been found in induced-CKD rat models [40] and in human AKI patients [39]. In order to further investigate genes involved in the NAD^+^ biosynthetic pathway, we performed gene set enrichment analysis (GSEA) on the differentially expressed gene (DEG) sets from the RNA-seq data (Fig. 4B, C). Expressions of many of the key genes that play central roles in intracellular NAD^+^ biosynthesis, such as *Nmnat1, Naprt*, and *Qprt*, were downregulated in CS kidneys (Supplementary Fig. 5). Interestingly, *Nampt* and *Bst1*, which are involved in the NAD^+^ salvage pathway to recycle NAD^+^ from nicotinamide (NAM), nicotinic acid (NA), nicotinamide riboside (NR), and nicotinamide mononucleotide (NMN) to maintain the cellular NAD^+^ levels, were increased. Also, the mRNA expression of *Nmnat2*, which converts cytosolic NAD^+^ from NMN and ATP, is upregulated in both CSA and CSB kidneys. In contrast, we found decreased expression of *Nmnat1* and *Nmnat3*, which are the nuclear and mitochondrial counterparts (Supplementary Fig. 5).

**Fig. 5:**
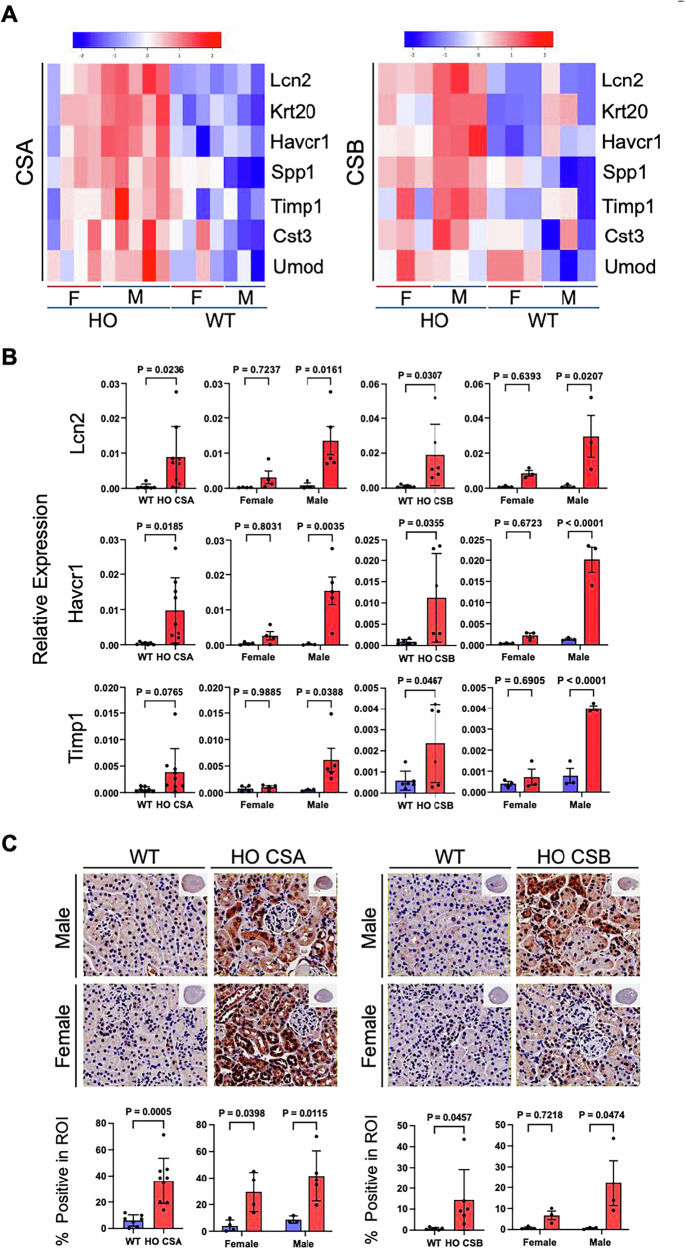
CS kidneys have increased expression of toxicity markers. **A** mRNA expression heatmap of the kidney injury markers (fold change ≥1.5-fold) in CS mice compared to WT mice. **B** qPCR was performed with the purified RNA from CS mice kidneys for kidney injury markers (*Lcn2*, *Havcr1*, and *Timp1*). *Actin* normalized expression values are shown. **C** Havcr1 immunohistochemistry (IHC) was performed with CS kidney tissue sections. A representative IHC image is shown (top), with quantitative analysis (bottom). Scale bar, 20 μm. Data are presented as mean ± SD (*n* ≥ 3). An unpaired t test was used for comparing CS with WT. A two-way ANOVA Sidak test was used for male vs female comparisons. *p* values indicated (Blue bar, WT; Red bar, HO CSA/CSB).

RNA-seq analysis also revealed significantly increased mRNA expression of kidney injury markers including *Lcn2*, *Krt20*, *Havcr1*, *Spp1*, *Umod*, *Timp-1*, and *Cst3* (Fig. 5A, B, and Supplementary Fig. 6). These are highly specific and sensitive renal injury markers that are used to detect early nephrotoxicity in drug development [41, 42]. Notably, induction levels of these injury markers are more obvious in the CS mouse kidney than in serum (Fig. 3I, J) and urine (Fig. 3K–N) analysis. Immunohistochemistry (IHC) analysis of the kidney sections stained with Havcr1 antibody revealed results consistent with the RNA-sequencing data, showing accumulated Havcr1 signals in both CSA and CSB knockout mice (Fig. 5C). Again, induction of both mRNA and protein expression (IHC) of these kidney injury markers were more prominent in male mice than in females.

#### Decreased Qprt expression in CS mouse models

**Fig. 6:**
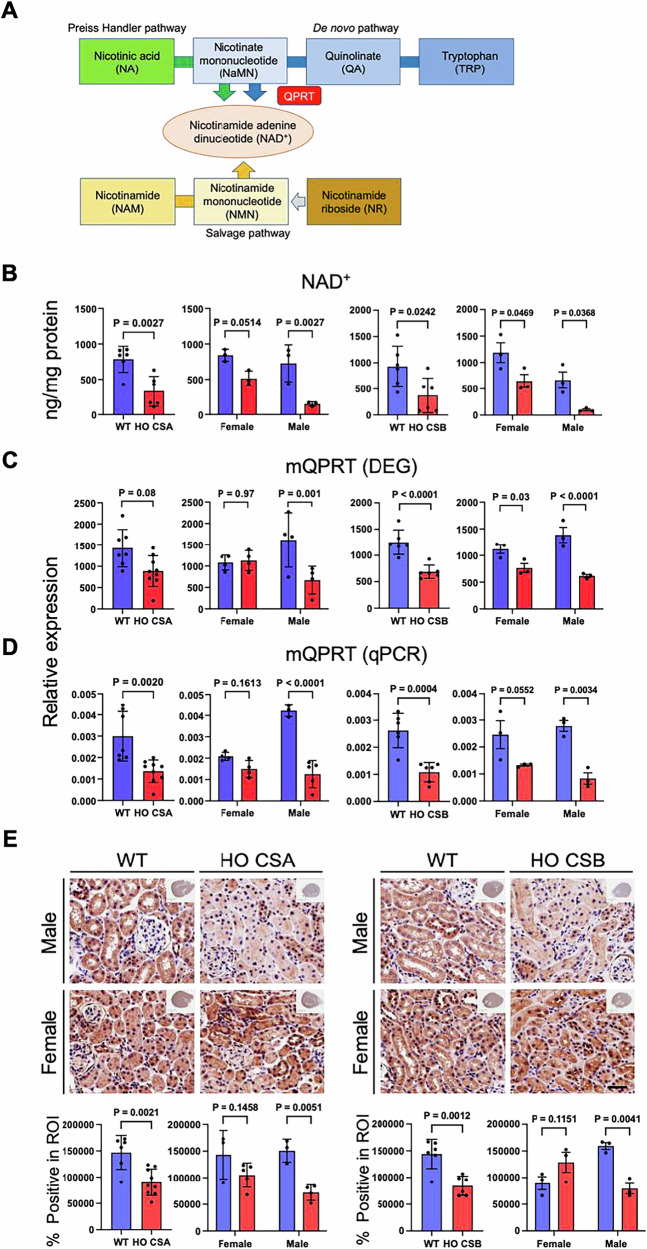
*mQPRT* expression is downregulated in CS mice kidneys. **A** Schematic diagram of intracellular NAD^+^ biosynthesis pathway in the kidney. The source of intracellular NAD^+^ comes from three pathways, Preiss-Handler (green), de novo (blue), and salvage pathways (yellow). **B** Total NAD^+^ quantification from the kidneys of CS and WT mice (*n* = 3). **C** *mQPRT* (mouse isoform) mRNA expression data from CSA and CSB mice kidney DEGs (**D**) *mQPRT* qPCR analysis with RNAs purified from WT/CS mice kidneys. *Actin* normalized expression values are shown. **E** mQPRT expression in kidney sections of CSA (left) and CSB (right) mice. Representative images for mQPRT IHC for CS mice for both sexes are shown. Scale bar, 20 μm. Data are presented as mean ± SD (*n* ≥ 3). An unpaired *t* test was used for comparing CS with WT. A two-way ANOVA Sidak test was used for male vs female comparisons. *p* values indicated (Blue bar, WT; Red bar, HO CSA/CSB).

Based on our RNA-seq analysis, *Qprt* was one of the most downregulated genes in both CSA and CSB kidneys (Fig. 4B, C). *Qprt* is a rate limiting enzyme in de novo biosynthesis of renal NAD^+^, playing a critical role in maintaining kidney function [43] as well as mediating resistance to acute kidney injury in aged populations [39] (Fig. 6A). Moreover, the kidney is one of the organs with the highest levels of both cellular NAD^+^ [44] and *Qprt* expression in humans and mice [45, 46]. Cellular NAD^+^ control is critical for maintaining renal metabolic and bioenergetic homeostasis since the renal tubular cells require a high level of energy for the filtrate reabsorption mediated by energy-intensive electrochemical gradients established across the apical and basolateral membranes of tubular epithelial cells [47]. Thus, NAD^+^ deprivation due to *Qprt* downregulation could cause a serious problem in renal function in CS patients. We measured the NAD^+^ levels in mouse kidney tissues and found significantly decreased NAD^+^ in CS kidneys (Fig. 6B and Supplementary Fig. 7). The decreased NAD^+^ levels were also reflected in the decreased NAD/NADH levels in CS kidneys (Supplementary Fig. 7). Next, we confirmed the involvement of *Qprt* in the pathology of CS kidneys by directly measuring *Qprt* expression levels. Indeed, we found significantly decreased *Qprt* mRNA expression in both CSA and CSB knockout mice, especially in males (Fig. 6C, D) except for CSA females, which coincide with the lack of changes in tryptophan metabolism-related gene expression changes (Fig. 4A). *Qprt* protein expression levels were verified by staining kidney sections with Qprt specific antibody, which showed significantly downregulated protein expression in kidneys of male CSA and CSB mice (Fig. 6E). These data correspond with the decreased mRNA expression of *Qprt* (Fig. 6C), which likely contributes to the lower NAD^+^ in CS mice kidneys.

#### Increased ATF3 stabilization in CS models

**Fig. 7:**
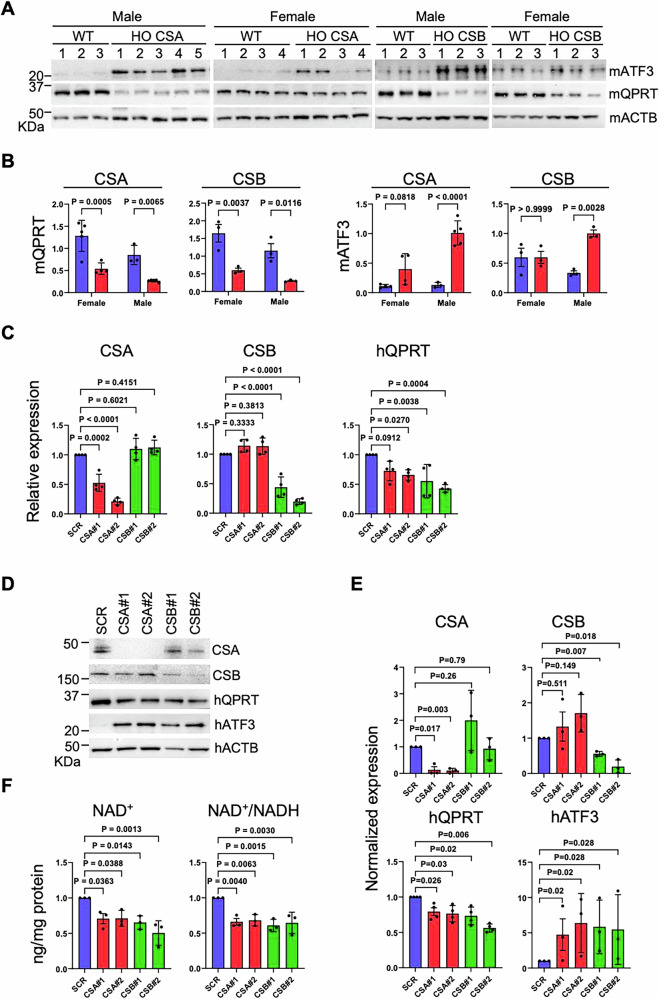
CSA/B availability determines QPRT expression. **A** CS mice kidney tissues were subjected to western blot for probing indicated antibodies. **B** Actin normalized mATF and mQPRT blot quantification data are shown (Blue bar, WT; Red bar, HO CSA/CSB). **C** HK-2 cells were transfected with two sets of CSA/B siRNAs. Cells were harvested 72 h post-transfection and subjected to qPCR. *GAPDH*-normalized expression values and (**D**) western blot analysis with indicated antibodies are shown. **E** Actin normalized blot quantification of CSA, CSB, hQPRT (human isoform) and hATF3 are shown. **F** siRNA-transfected HK2 cells were harvested and subjected to total protein-normalized NAD^+^ and NAD^+^/NADH quantification analyses. Data are presented as mean ± SD (*n* ≥ 3). An unpaired *t* test was used for comparing CS with WT and HK-2 Knockdown vs SCR. A two-way ANOVA Sidak test used for male vs female comparisons. A Mann–Whitney test used for ATF3 quantification. *p* values indicated.

Based on our data from CS mouse tissues, it is likely that decreased *Qprt* transcription results in an impaired de novo NAD^+^ synthetic pathway, which may cause chronic NAD^+^ deprivation leading to altered renal function in these mice. Therefore, we hypothesize that a CSA or CSB mutation would directly lead to these changes in renal tubular cells. It is reported that CSA and CSB dysfunction impairs the proteasomal degradation of genotoxic stress-induced overexpression of transcription repressor, activating transcription factor 3 (ATF3) [48]. Thus, in the absence of CS proteins, ATF3 retains the protein stability on its binding site and the expression of its responsive target genes are abrogated. In order to confirm ATF3 stabilization, we subjected CS kidney tissue samples to western blot. A significant increase in ATF3 protein level in both genotypes of CS (*CSA*^*−/−*^ *and CSB*^*m/m*^) male mice was observed while an increased trend was observed in CSA female compared to WT (Fig. 7A, B and Supplementary Fig. 8A). Also, in good alignment with the mRNA expression data (Fig. 6C, D), we found significantly downregulated Qprt protein expression in both CSA and CSB knockout mice, again more prominent in males. These data confirmed that ATF3 protein levels inversely correlating with Qprt expression levels in these animals (Fig. 7B).

Using a proximal tubular cell line (HK-2), derived from normal human kidney, we tested if the absence of CSA and CSB could recapitulate our findings from mouse kidney tissues. For this, we introduced two sets of siRNAs targeting CSA and CSB to knockdown endogenous CSA and CSB, respectively. As expected, both CSA and CSB knockdown caused significant *QPRT* transcription downregulation (Fig. 7C). Also, we found decreased QPRT and increased ATF3 protein levels when CSA or CSB was depleted (Fig. 7D, E and Supplementary Fig. 8B). Importantly, the concentration of intracellular NAD^+^ was significantly decreased in the cells when CSA or CSB was downregulated (Fig. 7F). These data confirm our findings from CS mouse kidney tissues, demonstrating that CSA and CSB regulated ATF3 stability may contribute to QPRT expression, which maintains intracellular NAD^+^ homeostasis.

#### ATF3 regulates QPRT expression in CS

**Fig. 8:**
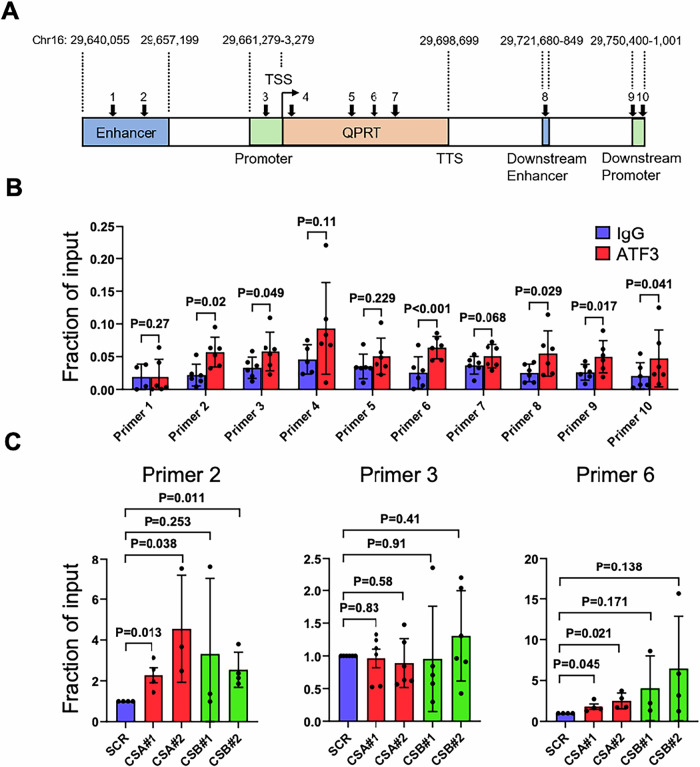
Direct interaction of ATF3 on the *QPRT* gene locus. **A** Schematic representation of *hQPRT* gene with potential hATF3 binding sites (marked with arrows). **B** Anti-ATF3/IgG chromatin immunoprecipitation (ChIP) was performed with HK-2 cells. Ten primer sets for respective potential hATF3 binding sites were investigated. **C** CSA/B siRNAs were transfected to HK-2 cells. 72 h post-transfection, hATF3 ChIP-qPCR was performed on *hQPRT* genomic locus. Data are presented as mean ± SD (*n* = 3). A paired *t* test used for analyses. *p* values indicated.

Next, we tested if the impaired *QPRT* expression was directly caused by persistent ATF3 interaction with its regulatory region. For this, we used a computational transcription factor binding site prediction tool (TRAP) [49] to investigate if ATF3 binding sites are present on the *QPRT* gene locus. There were several potential ATF3 interaction sites along the *QPRT* genomic locus (Fig. 8A), and primer sets were designed for ATF3 chromatin immunoprecipitation (ChIP) analysis (black arrows). To find the most potent ATF3 binding site, we performed a pilot ChIP experiment using ATF3 antibody and IgG control with quiescent HK-2 cells (Fig. 8B). Out of 10 candidate sites, we found significant induced ChIP signal over IgG on several sites along the *QPRT* gene, which includes upstream enhancer (#2), proximal promoter (#3), gene coding region (#6), downstream enhancer (#8), and downstream distal promoters (#9 and #10). Among these candidates, three in the upstream regulatory region and one in the gene coding region were further analyzed under CS-deprived conditions (Fig. 8C). CSA knockdown significantly increased ATF3 occupancy on both the upstream enhancer (Primer 2) and gene coding region (Primer 6), and CSB knockdown was effective on the upstream enhancer (Primer 2) locus (Fig. 8C), reflecting the direct ATF3 occupancy control by CS proteins. However, CSA and CSB knockdown did not change ATF3 interaction on the promoter region (Fig. 8C, Primer 3). These data support our hypothesis that CS protein availability affects ATF3 occupancy on the QPRT gene locus to regulate mRNA transcription.

#### NAD^+^ supplementation ameliorates kidney injury and reduces apoptosis in CSA/CSB knockdown tubular cells

**Fig. 9:**
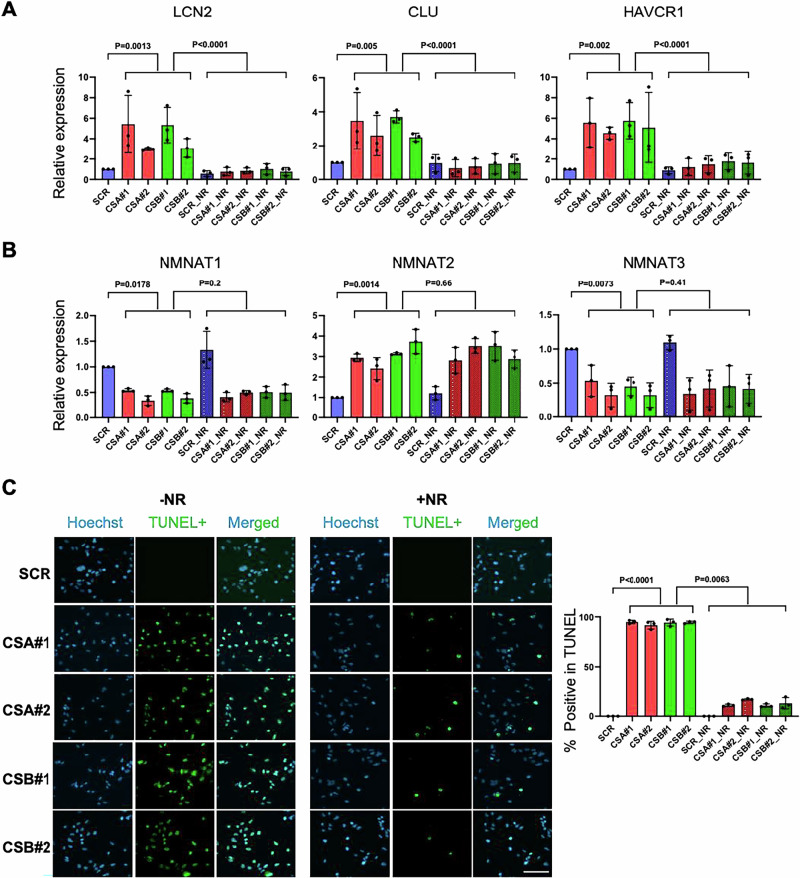
NAD supplementation mitigates kidney injury and apoptosis in CSA/CSB knockdown HK-2 cells. HK-2 cells transfected with two sets of CSA/B siRNAs and treated with 1 mM NR for 24 h. Cells were harvested after NR treatment and subjected to qPCR and TUNEL staining. mRNA expression for (**A**) kidney injury markers *LCN2*, *CLU*, and *HAVCR1* and (**B**) *NMNATs 1,2,3* were analyzed. *ACTIN* normalized expression values are shown. **C** TUNEL staining was performed on cells with/without NR treatment. A representative image (left) is shown with quantitative analysis (right). Scale bar, 100 µm. An unpaired *t* test was used to compare HK-2 Knockdown vs SCR with/without NR treatment.

To model kidney features in CS mice, we first examined the expression of kidney injury markers in HK-2 cells lacking CSA and CSB. Successful knockdown efficiency was confirmed by western blot (Supplementary Fig. 9). The kidney toxicity markers *Lcn2, Clu*, and *Havcr1* were significantly elevated in CSA/CSB knockdown HK-2 cells compared to controls. To further investigate the role of NAD^+^ in kidney function, we supplemented with nicotinamide riboside (NR) in CSA and CSB knockdown HK-2 cells. NR supplementation significantly reduced the expression of these toxicity markers, suggesting that NR can mitigate the effects of NAD^+^ depletion in CSA/CSB knockdown cells (Fig. 9A). The expression patterns of NAD^+^ salvage pathway enzymes *NMNAT1*, *NMNAT2*, and *NMNAT3* in CSA/CSB knockdown HK-2 cells mirrored those observed in CSA/CSB mouse models (Fig. 9B). Specifically, we observed decreased expression of *NMNAT1* and *3*, along with increased *NMNAT2* mRNA levels, none of which were altered by NR supplementation. To examine the impact of NR on apoptosis, we performed a TUNEL assay. Apoptosis levels were significantly higher in CSA/ CSB knockdown HK-2 cells, whereas NR treatment markedly reduced apoptosis (Fig. 9C). These findings suggest that NR supplementation may mitigate kidney injury by restoring intracellular NAD^+^ levels and reduce apoptosis.

## Discussion

PARP1 hyperactivation and lower intracellular NAD^+^ have been well demonstrated in cells derived from CS patients and animal models such as mice and worms. However, in animal models, PARP1 inhibition does not recover all the features of CS, including mitochondrial functions, while NAD^+^ supplementation has a strong effect on improving CS [4]. This indicates the presence of additional mechanisms, besides PARP1 hyperactivation, that contribute to intracellular NAD^+^ deprivation. Moreover, despite the advances in understanding its biochemical characteristics, the molecular pathogenesis of many key CS features, including kidney dysfunction, is not fully understood.

In the present study, we investigated kidney pathology in CSA/B knockout mice. While renal failure is reported to have a major influence on the prognosis of CS patients, the symptoms present only after substantial kidney damage has already occurred, making it futile to intervene therapeutically at this late stage. Thus, a more comprehensive investigation into CS-related kidney dysfunction is needed to improve CS pathophysiology and with the goal of developing interventions for renal dysfunction.

The key finding in our study is that the renal failure in CS may be caused by a disrupted intracellular NAD^+^ biosynthetic system. This is supported by our findings of decreased expression of *Qprt* in CS kidneys, which is a key enzyme in maintaining intracellular NAD^+^ homeostasis. ATF3 is a transcription repressor that is known to play a central role in transcription repression after DNA damage in CS cells [48], and we showed increased ATF3 protein expression in the absence of CS proteins, which may be responsible for the observed Qprt impairment in CS kidneys.

A decreased GFR rate was observed in 45% of CS patients [19] and some kidney lesions reported in CS patients [31, 50] are also found in our CS mice. The lesions observed in kidney tissues of CS male mice were located predominantly in tubular epithelial cells and resemble the CPN found in aged rats [30] and tubular atrophy seen in CS patients [31]. In line with this, we observed increased BUN, creatinine, and Cst3 levels in CS mice, which reflects the reduced GFR rate in these animals (Fig. 3). One of the characteristic features of human CS patients is muscle atrophy. CS mice also show reduced muscle strength [10] and cachexia [51], which are possible indications of muscle wasting. Since serum creatinine levels closely correlate with muscle mass, lower basal serum creatinine levels in CS mice are expected because of smaller muscle mass. However, the increased serum creatinine level we found in CS mice may reflect facilitated muscle degeneration in these animals (Fig. 3B). High urine calcium levels can contribute to and indicate tubular injury in the kidneys [52]. Thus, the elevated urine calcium we see in CS mice (Table 1) may reflect tubular injury in CS mice kidneys. Importantly, despite all the evidence of renal functional impairment we observed in our study, we did not detect proteinuria in CS mice (Table 1). Investigating the protein component more in depth in CS mice urine will provide more detailed information.

**Fig. 10:**
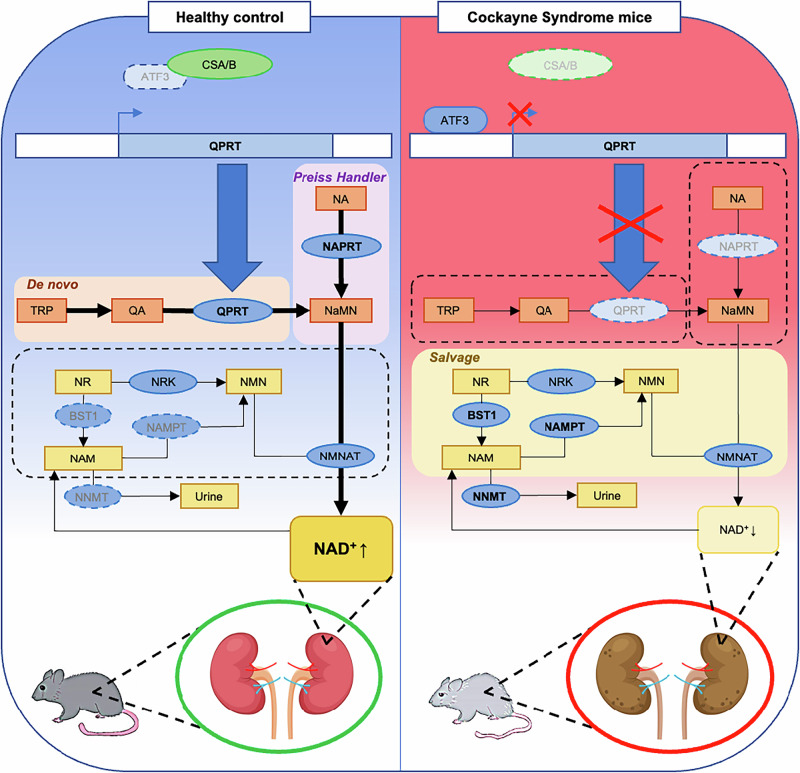
Potential mechanism of renal dysfunction in CS mice. In healthy animals, renal tubular cells utilize high-demand NAD^+^ supplies from Preiss-Handler and de novo synthetic pathways by metabolizing nicotinamide (NA) and tryptophan (TRP), respectively. In the case of CS protein disruption (CS), increased ATF3 expression suppresses *QPRT* transcription, leading to an impaired de novo pathway. Moreover, decreased nicotinate phosphoribosyltransferase (NAPRT) expression may also lead to the Preiss-Handler pathway blockage. Impairment of both Preiss-Handler and de novo NAD^+^ synthetic pathways result in overall intracellular NAD^+^ deprivation, which may subsequently lead to kidney damage.

Renal proximal tubules contain a large number of mitochondria to support the energy-demanding process of water and solute reabsorption. In the renal cortex, a significant amount of ATP is generated by the mitochondrial TCA cycle and β-oxidation [53], and low NAD^+^ levels and impaired mitochondrial ETC lead to mitochondrial ROS production [54, 55]. We have reported that CSA and CSB depletion led to decreased intracellular NAD^+^ due to persistent chromatin PARylation [4]. Therefore, it is very likely that renal cells in CS patients readily experience insufficient intracellular NAD^+^. In addition to this, compromised Qprt expression may exacerbate intracellular NAD^+^ deprivation, and subsequently, the lower NAD^+^ may contribute to cellular damage and ultimately to the development of renal disease (Fig. 10). In our experiments, CSA/B knockdown human kidney tubular epithelial cells exhibited a significant increase in TUNEL staining intensity, which was markedly suppressed by NAD⁺ supplementation with NR (Fig. 9C).

Our histopathological analysis of tissue samples from young (17–21 weeks) and aged (45–68 weeks) CS mice revealed that tubular injuries were more pronounced in younger animals (Fig. 2A), while aged animals exhibited additional glomerular damage including podocyte loss (Fig. 2B, D, E, I). Given that podocytes primarily rely on anaerobic glycolysis and are relatively resistant to mitochondrial stress [56, 57], this supports the notion that NAD⁺ insufficiency may contribute to tubular injury from an early age, rather than direct glomerular damage. As tubular damage accumulates, persistent stress—such as altered glomerular hemodynamics, activation of pro-inflammatory and fibrotic signaling pathways, and glomerular hypertension and hyperfiltration—may ultimately lead to podocyte damage and glomerular injury. Supporting our model, a previous report also showed that QPRT downregulation is associated with renal injury and progression from AKI to CKD [58]. Moreover, loss of de novo NAD^+^ synthetic pathway enzymes is associated with renal anomalies in humans and mice due to insufficient NAD^+^ for proper development [59]. It has been shown that PGC1α [60] and ACMSD [61] regulated NAD biosynthesis provide renal protection against injury and mitochondrial homeostasis maintenance in kidney, respectively.

In CS mice tissue sections and CSA/B knockdown HK-2 cells showed a significant increase in TUNEL staining intensity (Fig. 2J and Fig. 9C). In HK-2 cells, this effect was largely suppressed by NAD^+^ supplementation with NR, suggesting that NAD^+^ deprivation in CSA/B knockdown cells promotes apoptotic cell death. Since low intracellular NAD^+^ levels and mitochondrial ETC dysfunction are major contributors to intracellular ROS production [54], the resulting ROS accumulation may have activated cell death pathways. In addition, intracellular NAD^+^ depletion disrupts ATP production and plasma membrane homeostasis, leading to apoptosis, autophagy, and oncosis [32]. While ferroptosis is a well-established mechanism of tubular epithelial cell death under ischemia-induced hypoxic renal stress [62], its role in intracellular NAD^+^ deprivation remains uncertain. Both CSA and B proteins are involved in the repair of oxidative DNA lesions in the nucleus and mitochondria, and are also essential for TC-NER in the nucleus, which is responsible for eliminating lesions in the transcribed strand of active genes [63]. Thus, in addition to the insufficient NAD^+^-driven ROS generation, impaired DNA damage repair due to the absence of CS proteins, may also have caused increased accumulation of oxidative DNA damage to the kidney cells. Although we have detected enhanced apoptosis in CSA/B knockdown human tubular cells and CS animals, the involvement of other cell death pathways, such as ferroptosis or necroptosis, cannot be excluded. A comprehensive exploration of the precise mechanism of cell death falls beyond the scope of the present study but represents an interesting direction for future research.

Mouse studies using isotope-tracer methods to investigate the NAD flux quantitation analysis confirmed that the kidney has a net excretion of NAM made from both tryptophan and NA [64]. This suggests that the source of NAD^+^ in kidney is predominantly from Preiss-Handler and de novo biosynthesis pathways. We found decreased expression of *Naprt*, along with *Qprt* (Supplementary Fig. 5) in CS kidneys. This suggests that the high NAD^+^ demand in CS kidneys may, in part, have caused an intracellular metabolic reprogramming to utilize the NAD^+^ salvage pathway rather than relying on Preiss-Handler and de novo biosynthesis pathways. This is done by overexpressing genes such as *Nampt* and *Bst1* (Fig. 4B and Supplementary Fig. 5) to possibly compensate for the insufficient intracellular NAD^+^ and to maintain renal homeostasis, while overexpressing *Nnmt* to clear out accumulating intracellular NAM for urinary secretion (Fig. 10). Intriguingly, we also found that the expression pattern of *Nmnat* isoforms were greatly altered in kidneys of CS mice (Supplementary Fig. 5). *Nmnat1*, *2*, and *3* catalyze the last step in the biosynthesis of NAD^+^ in the nucleus, cytosol, and mitochondria, respectively. Decreased expression of *Nmnat1* and *3* are in line with our findings of downregulated *Qprt* expression, demonstrating the impaired overall intracellular NAD^+^ biosynthesis pathway. Decreased expression of *Nmnat1*, *3*, and *Qprt* has also been found in the induced CKD rat model [40], which supports our findings. On the other hand, mRNA expression of *Nmnat2* is increased in CS mice kidneys, unlike its other isoforms. It has been shown that mRNA expression of *Nmnat2* in rat hearts is significantly upregulated during aging, which is likely a compensatory mechanism to maintain the cardiac level of NAD^+^ to fuel mitochondrial energy production [65]. It is also worth noting that in these rats, the basal level of *Nmnat2* expression was nearly undetectable in the kidneys. Thus, it is likely that the increased *Nmnat2* in CS mice kidneys may also reflect the existence of compensatory mechanisms to cope with an intracellular NAD^+^ shortage. Additionally, our experimental model using human kidney tubular epithelial cells mirrors these observations in animals (Fig. 9B). This finding aligns with previous reports suggesting that NMNAT2 is the most NAD^+^-sensitive isoform among the NMNAT family and may function as an intracellular NAD^+^ sensor [66]. In this context, further investigation into the effect of NAD^+^ supplementation on kidney pathology in CS animal models would be valuable.

Another remarkable finding in our study is that the male CS mice are showing much more severe and stronger kidney pathology than females (Fig. 1B, C, E). These findings were consistent in serum (Fig. 3), and in gene expression profiles (Figs. 4 and 5). These observations were also supported by our western blot data, where males had greater QPRT silencing and ATF3 stabilization (Fig. 7). This may be explained by our findings showing that male mice have a higher magnitude of intracellular NAD^+^ deprivation (Fig. 6B, ~60% reduction) compared to females (~30% reduction). In addition, the more prominent phenotype observed in CSA male mice could be reflected in the significantly increased serum phosphorous, chlorine and potassium levels than CSB males (Supplementary Fig. 3). In CKD, electrolyte disorders, such as hyperkalemia, are highly prevalent. One cause of hyperkalemia is decreased glomerular filtration rate and insufficient tubular capacity to secrete potassium [67]. While the calcium-phosphorous balance is maintained by proper functioning of the kidney, decreased kidney excretion of phosphate leads to hyperphosphatemia in CKD [68]. When GFR rates are decreased, the calcium and phosphate are released from bone causing bone demineralization, muscle weakness, atrophy, and calcification of soft tissues [68, 69]. In our CSA male mice, the body weight is significantly reduced compared to their CSB counterparts (Fig. 1A), which might be due to disrupted interplay between kidney calcium-phosphate homeostasis and bone metabolism. It is also reported that human males are more susceptible to kidney damage than human females, including both AKI [70] and CKD [71].

Studies on Cockayne Syndrome utilizing large human cohorts have reported a slightly higher representation of males (Wilson et al., 44 females and 58 males [72]; Nance et al., 60 females and 75 males [73]). However, due to the extreme rarity of CS, there is insufficient data to establish any statistically significant racial or sex-based predisposition regarding renal symptomatology or the syndrome itself [74]. While the exact mechanism behind this is still unclear, it is suspected that the development of kidney injury in males may be at least partially a result of the detrimental effects of androgens, such as testosterone [70]. While CS mice do not, in general, reflect many of the features seen in human CS patients, it is of particular interest that they do reflect this pathology. Interestingly, CS mice also recapitulate the hearing and vision loss seen in the patients [1, 73, 75] and this lends more credibility to the use of mice in studies on CS.

Overall, our study is expected to have a positive impact on relieving the pathological changes of CS disease. Additionally, it may contribute to a better understanding of the biology of premature aging and inform strategies aimed at prevention. Further, it may also inform the development of effective interventions to maintain health, well-being, and function and prevent or reduce the burden of age-related diseases.

## Supplementary information

Supplemental material

## Data Availability

The data and materials used in this study are available upon reasonable request from the corresponding author.The RNA-seq and datasets generated specifically for this study can be accessed from the NCBI’s Gene Expression Omnibus (GEO) repository (https://www.ncbi.nlm.nih.gov/geo/) using the accession number GSE246444.
