# Supplementary material for: Cockayne syndrome mice reflect human kidney disease and are defective in de novo NAD biosynthesis
Source: Cell Death Differ. 2025 May 15;32(11):2126–45. doi: 10.1038/s41418-025-01522-7 (PMC12572305; doi:10.1038/s41418-025-01522-7)
Supplement: Supplementary file 1 — Supplemental material [file 41418_2025_1522_MOESM1_ESM.pdf]

Cockayne syndrome mice reflect human kidney disease and are defective in *de novo* NAD biosynthesis

Komal Pekhale[1,2], Vinod Tiwari[1], Mansoor Hussain[1], Christy C. Bridges[3], Deborah L. Croteau[1,4], Moshe Levi[5], Avi Z. Rosenberg[6] , Briana Santo [7], Xiaoping Yang [6], Tomasz Kulikowicz[1] , Xiaoxin X. Wang[5], Jong-Hyuk Lee[1,3,8,*,#], and Vilhelm A. Bohr[1,9,10,*,#]

[1]DNA repair section, National Institute on Aging, National Institutes of Health, Baltimore, MD 21224;
[2]Cecil H. and Ida Green Center for Reproductive biology Sciences, University of Texas Southwestern Medical Center, Dallas, TX 75390;
[3]Department of Biomedical Sciences, Mercer University School of Medicine, Savannah, GA 31404;
[4]Computational Biology & Genomics Core, Laboratory of Genetics and Genomics, National Institute on Aging, National Institutes of Health, Baltimore, MD 21224;
[5]Biochemistry and Molecular & Cellular Biology, Georgetown University Medical Center, Washington, DC, United States.
[6]Department of Pathology Johns Hopkins University School of Medicine Baltimore, MD;
[7]Johns Hopkins University School of Medicine
[8]Center for Gerontology, Mercer University, Macon, GA 31207;
[9]Danish Center for Healthy Aging, University of Copenhagen, 2200 Copenhagen, Denmark;
[10]Lead Contact

*Both authors contributed equally.

#To whom correspondence should be addressed. Email: vbohr@sund.ku.dk or lee_jh@mercer.edu

Running Title: NAD deficiency in cockayne syndrome mice

Supplementary Data

Supplementary Figure 1

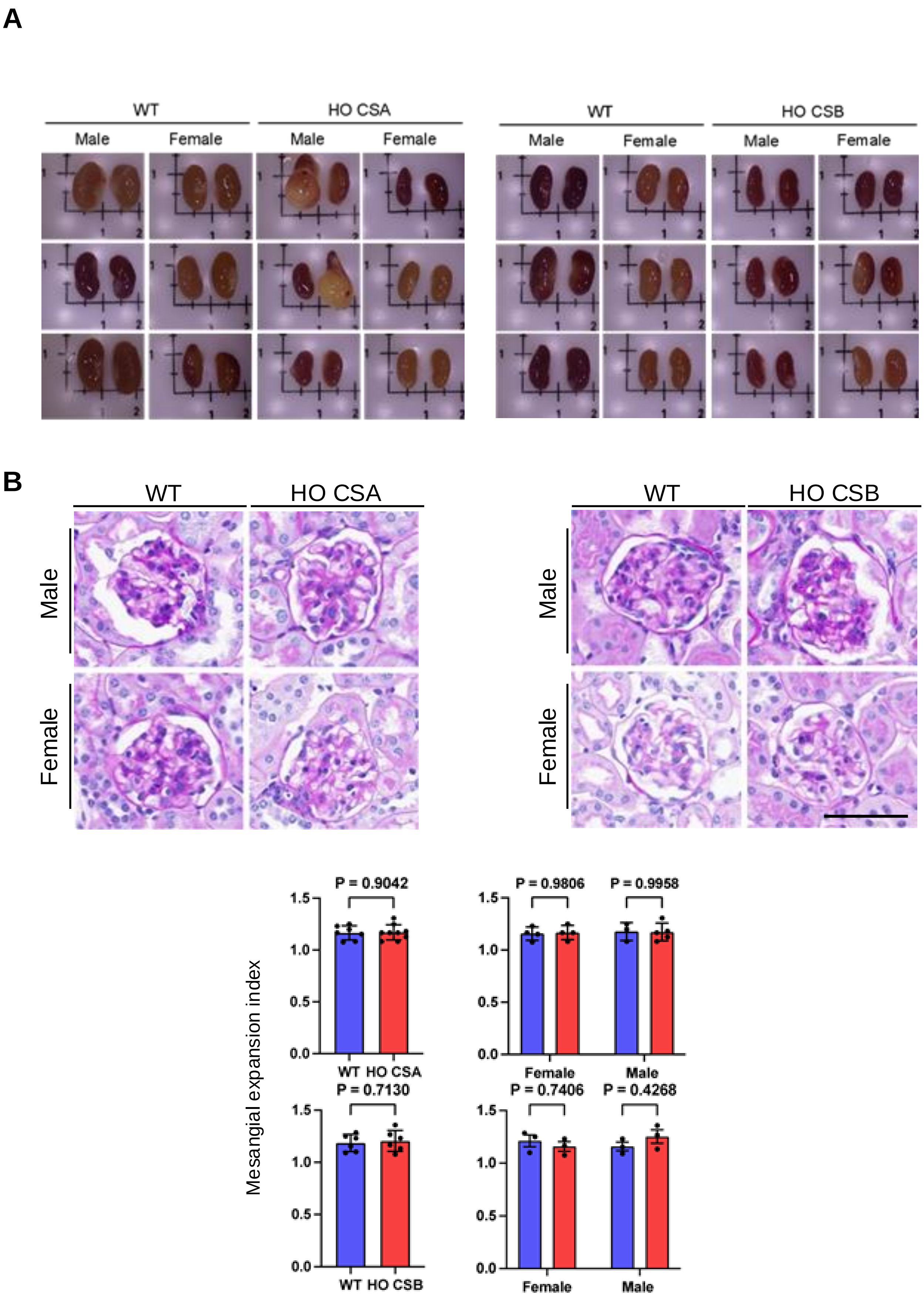

Supplementary Figure 1

(A) Kidneys were removed from 45-86 weeks-old mice at termination and directly compared. (WT= wildtype; HO=Homozygous; M= Male; F= Female) Background scale × 10 mm. (B) Representative image of PAS staining in CS mice. Scale bar, 50 µm. The mesangial expansion index is defined as the ratio of the mesangial area to the glomerular tuft area. The mesangial area is measured by assessing the PAS-positive, nucleus-free regions within the mesangium. Data are presented as mean ± SD (n≥3), An unpaired t-test was used for comparing CS with WT. A two-way ANOVA Sidak test was used for the comparisons between males and females. p-values indicated (Blue bar, WT; Red bar, HO CSA/CSB).

Supplementary Figure 2

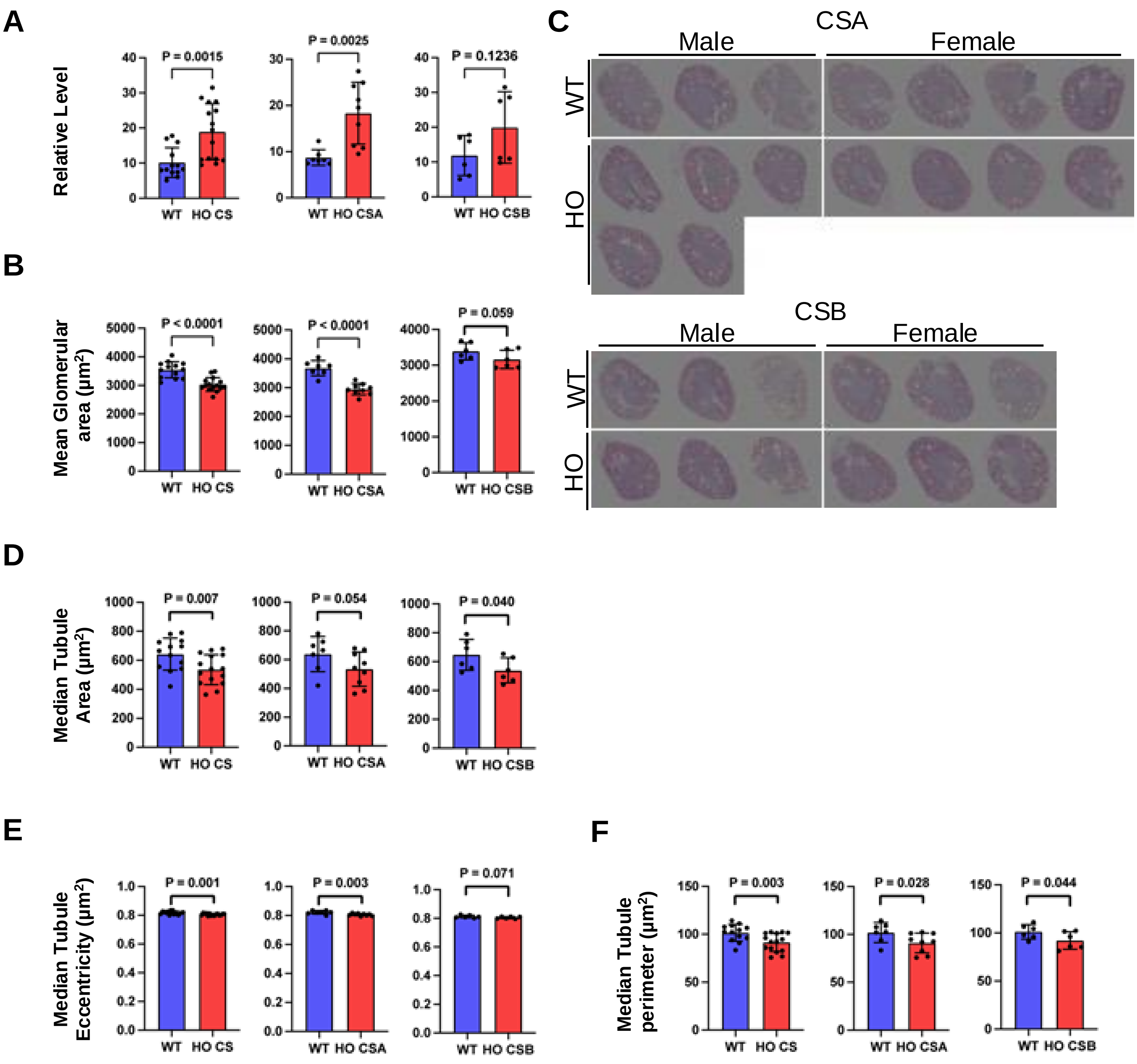

Supplementary Figure 2

(A) Quantification of PSR staining for WT vs CS , WT vs CSA , WT vs CSB. (B) Glomerulus quantification from whole slide H&E images (C) Glomerular area feature mapping. (D-F) Tubular feature quantifications (D) Median Tubular area, (E) Median tubule eccentricity, and (F) Median Tubule Perimeter by digital histology. Data are presented as mean ± SD (n≥3), Statistical analysis was performed as mentioned in materials and methods. p-values indicated (Blue bar, WT; Red bar, HO CSA/CSB, CS).

Supplementary Figure 3

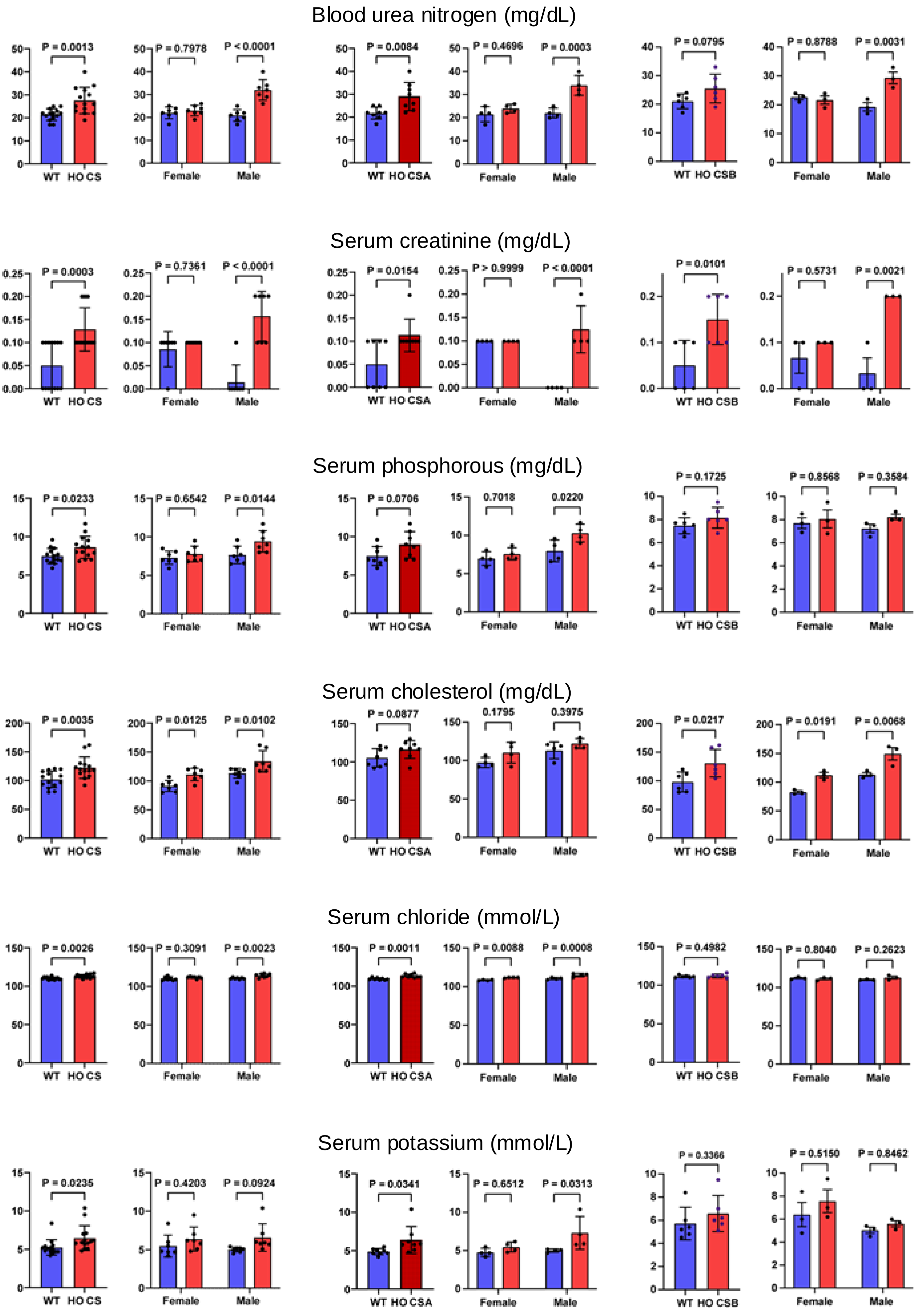

Supplementary Figure 3

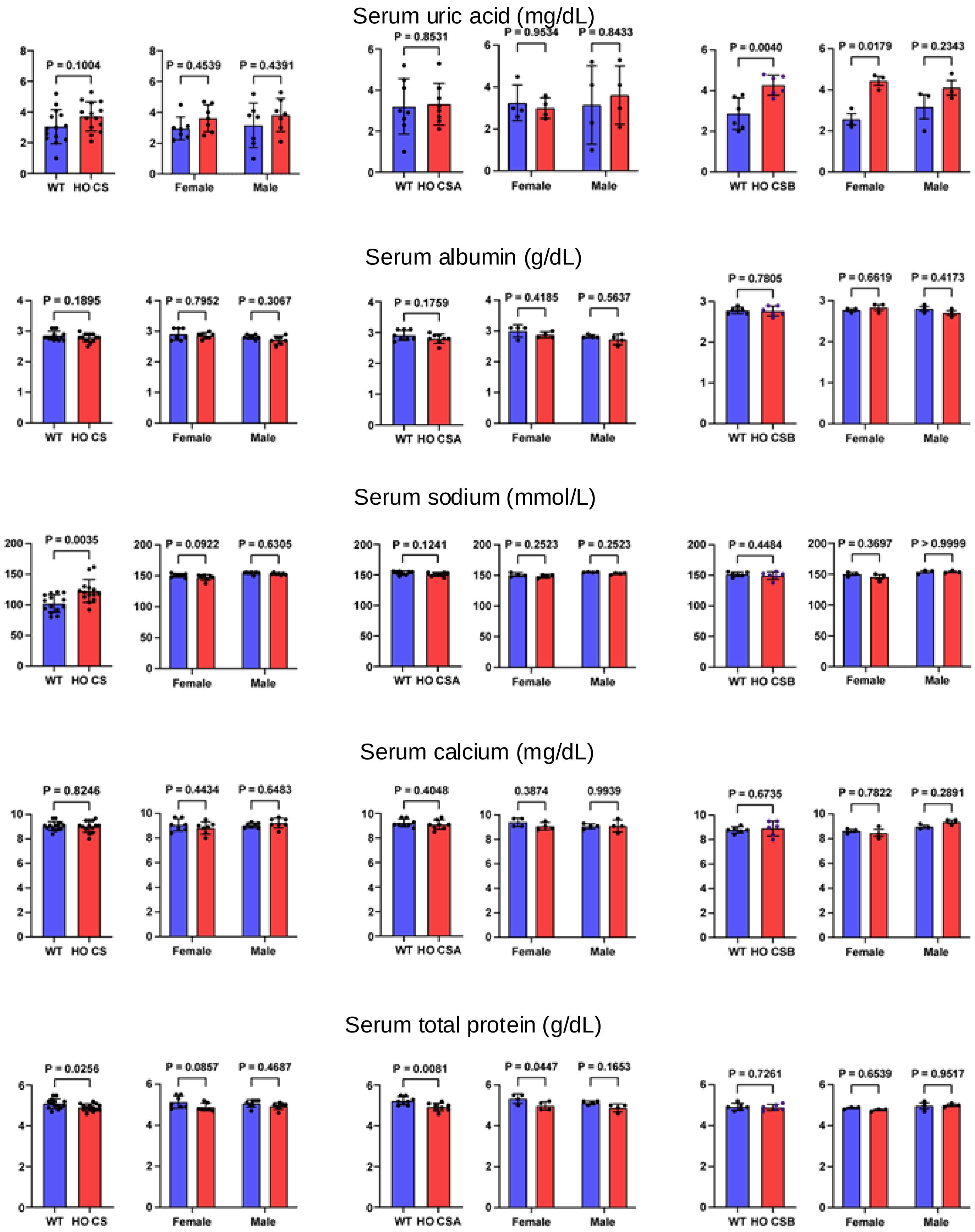

Supplementary Figure 3
Serum isolated from the whole blood samples of the WT and CS mice were collected and analyzed for the parameters indicated. Data presented as mean ± SD (N≥3), Unpaired t-test was used for comparing CS with WT. Two-way ANOVA test used for male vs female comparison. P-values indicated (Blue bar, WT; Red bar, HO CSA/CSB).

Supplementary Figure 4

A

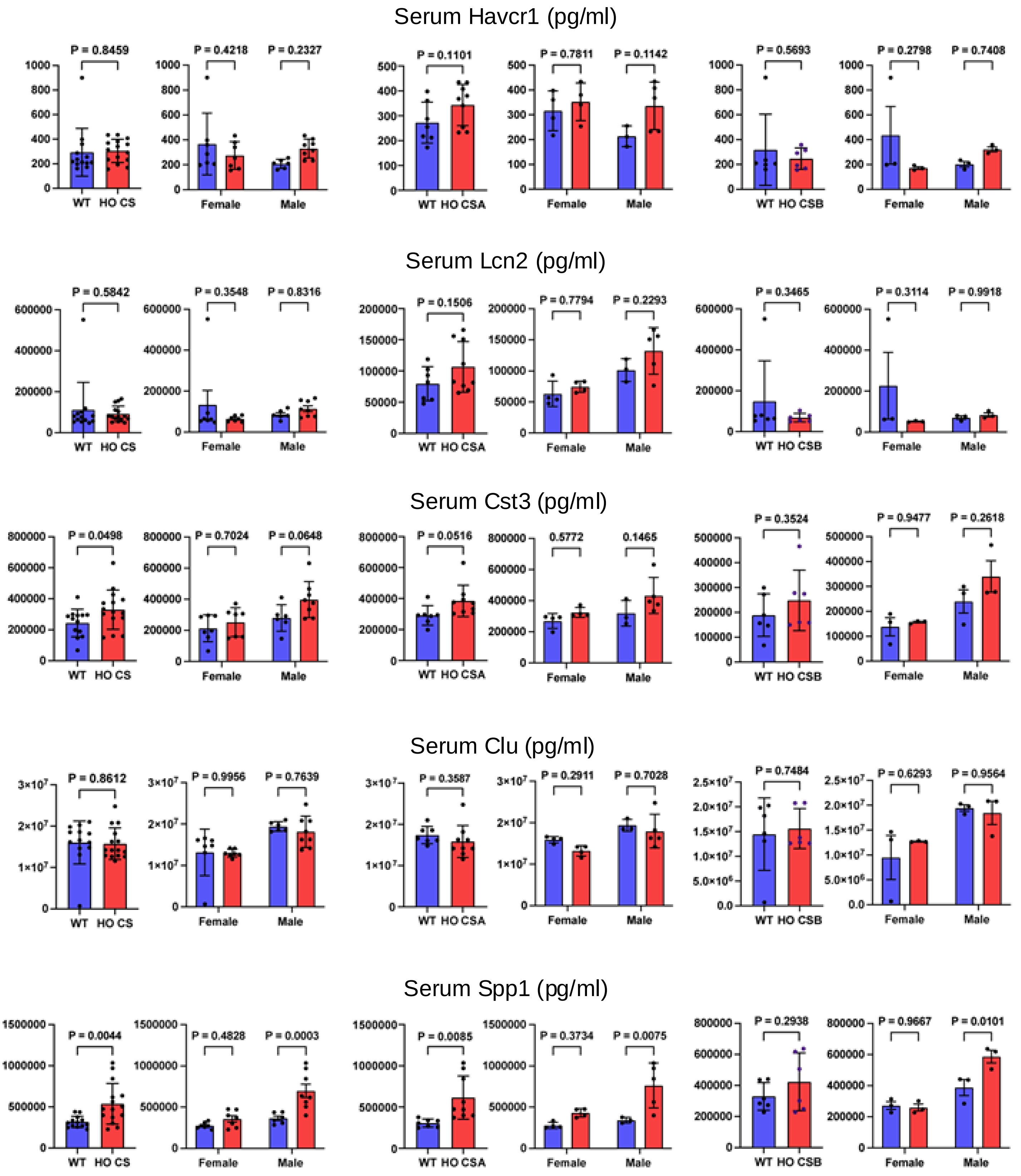

Supplementary Figure 4

B

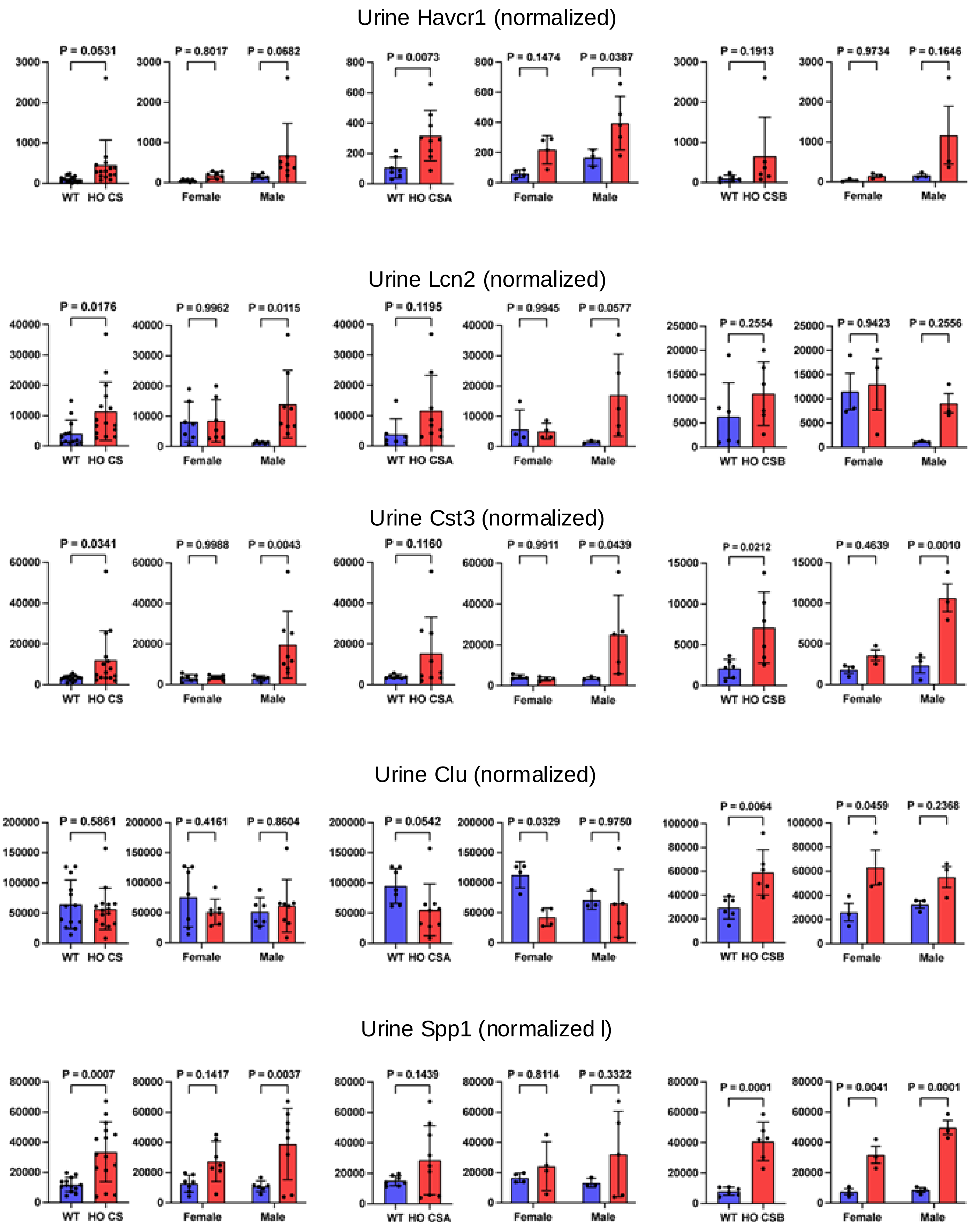

Supplementary Figure 4

Measurement of (A) kidney toxicity markers in serum and (B) Creatinine normalized urine values of CS mice. Data shown as mean ± SD (N≥3), Unpaired t-test was used for comparing CS with WT. Two-way ANOVA test used for male vs female comparison. P-values (Blue bar, WT; Red bar, HO CSA/CSB).

Supplementary Figure 5

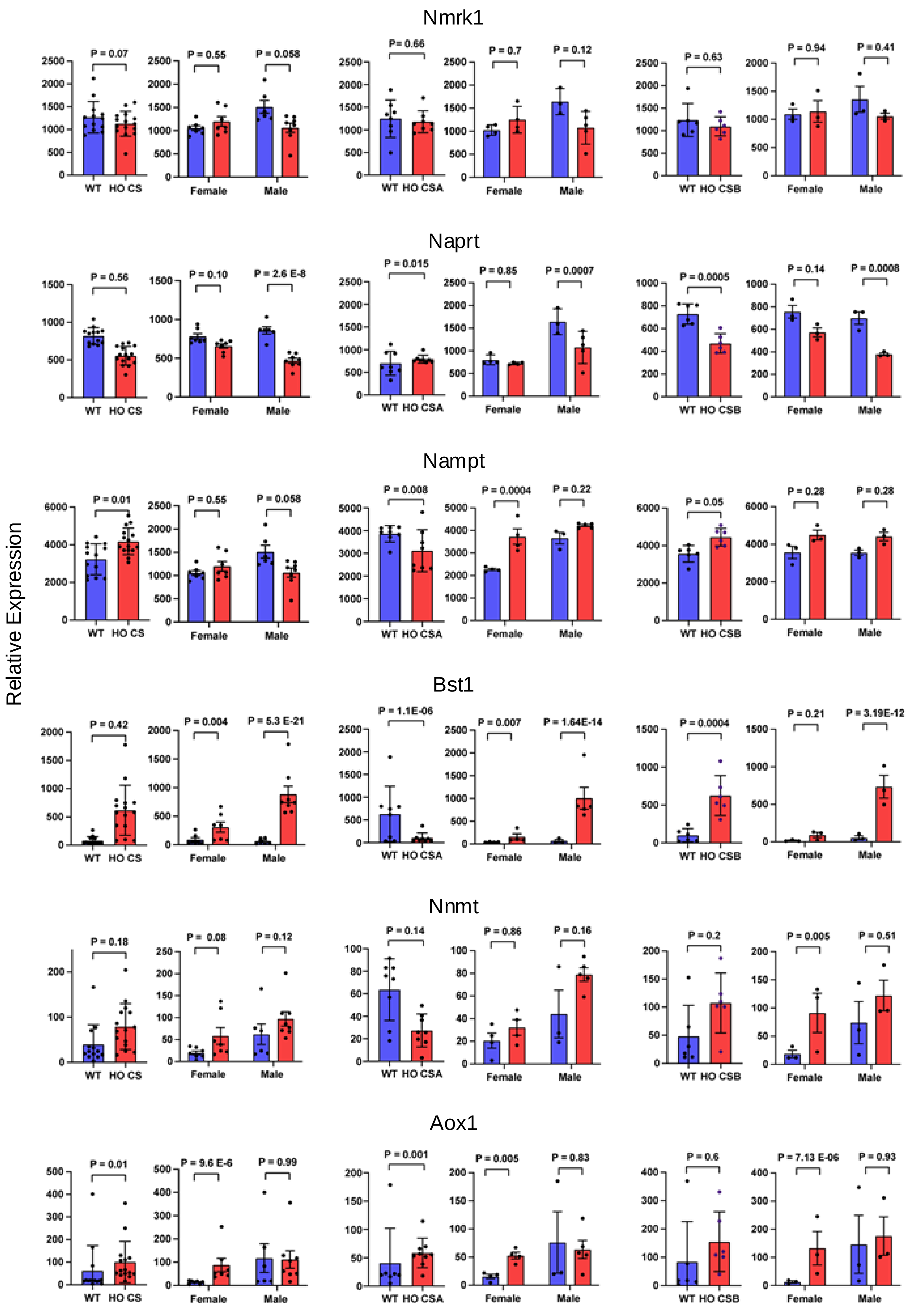

Supplementary Figure 5

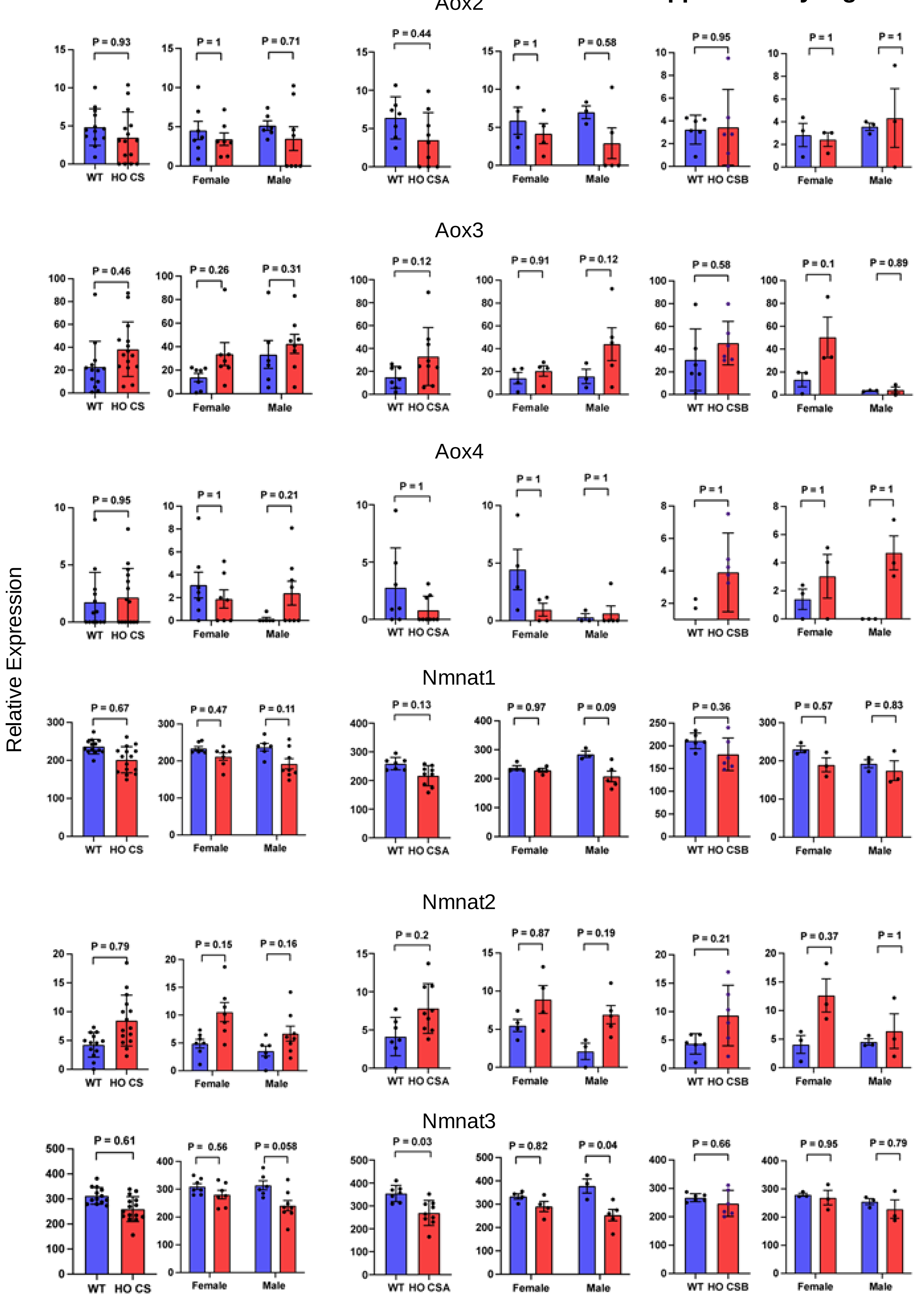

Supplementary Figure 5

Expression of genes in 'Nicotinate and Nicotinamide metabolism' panel from DEGs of WT and CS mice kidney RNA-sequencing analysis. P-adjusted values indicated (Blue bar, WT; Red bar, HO CSA/CSB).

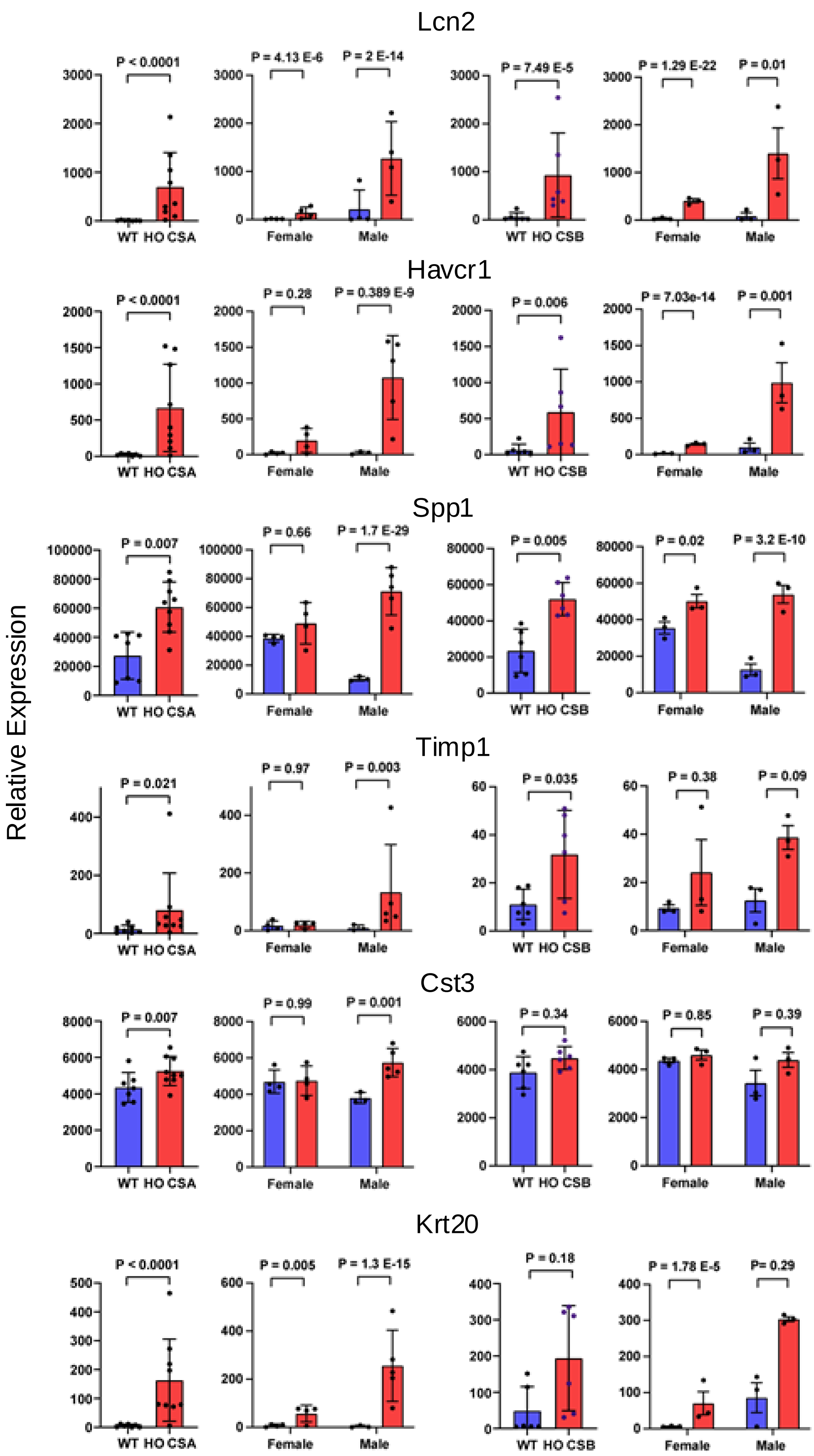

Supplementary Figure 6

Expression of kidney toxicity markers in kidney from DEGs of WT and CS mice kidney RNA-sequencing analysis. P adjusted-values indicated (Blue bar, WT; Red bar, HO CSA/CSB).

Supplementary Figure 7

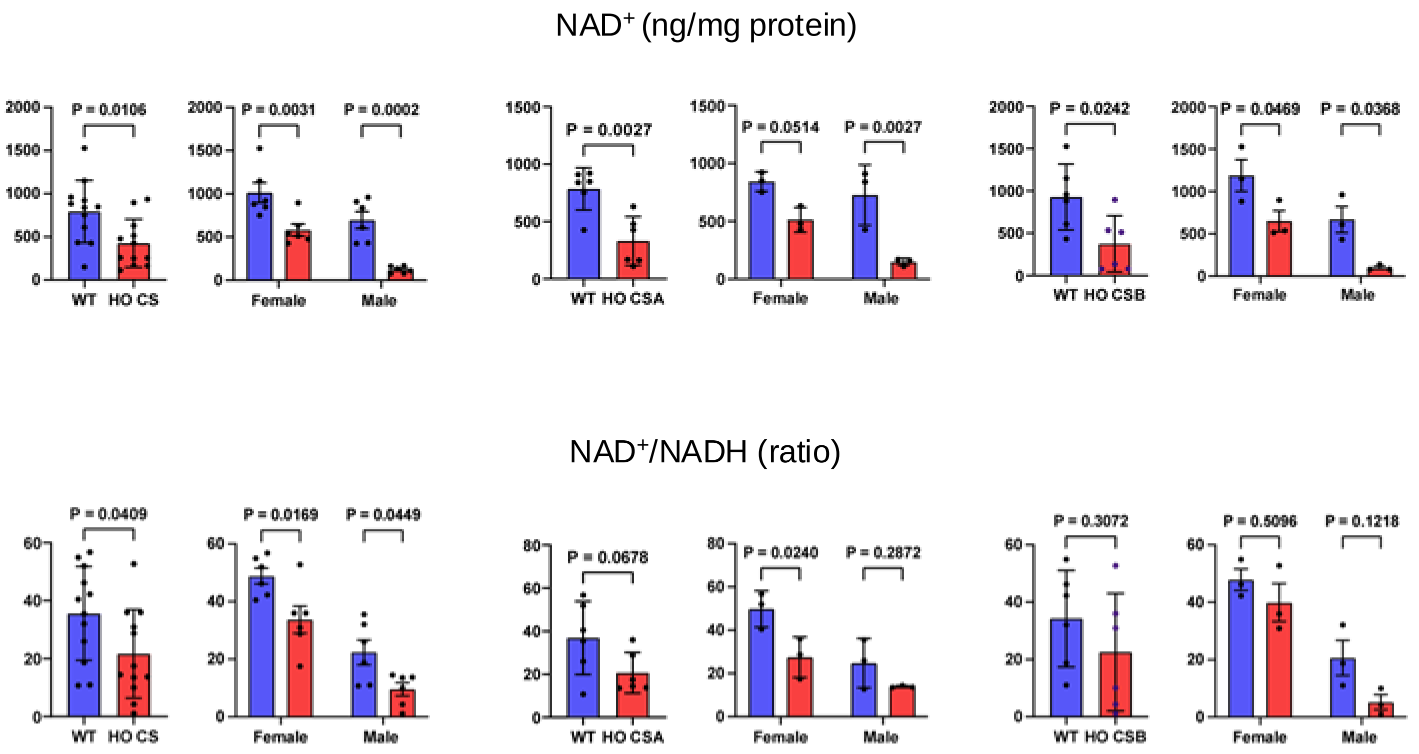

Supplementary Figure 7

Measurement of NAD$^+$ and NAD/NADH levels in WT and CS mice kidneys. Data are shown as mean ± SD (N≥3), Unpaired t-test was used for comparing CS with WT. Two-way ANOVA test used for male vs female comparison. P-values indicated (Blue bar, WT; Red bar, HO CSA/CSB).

Supplementary Figure 8

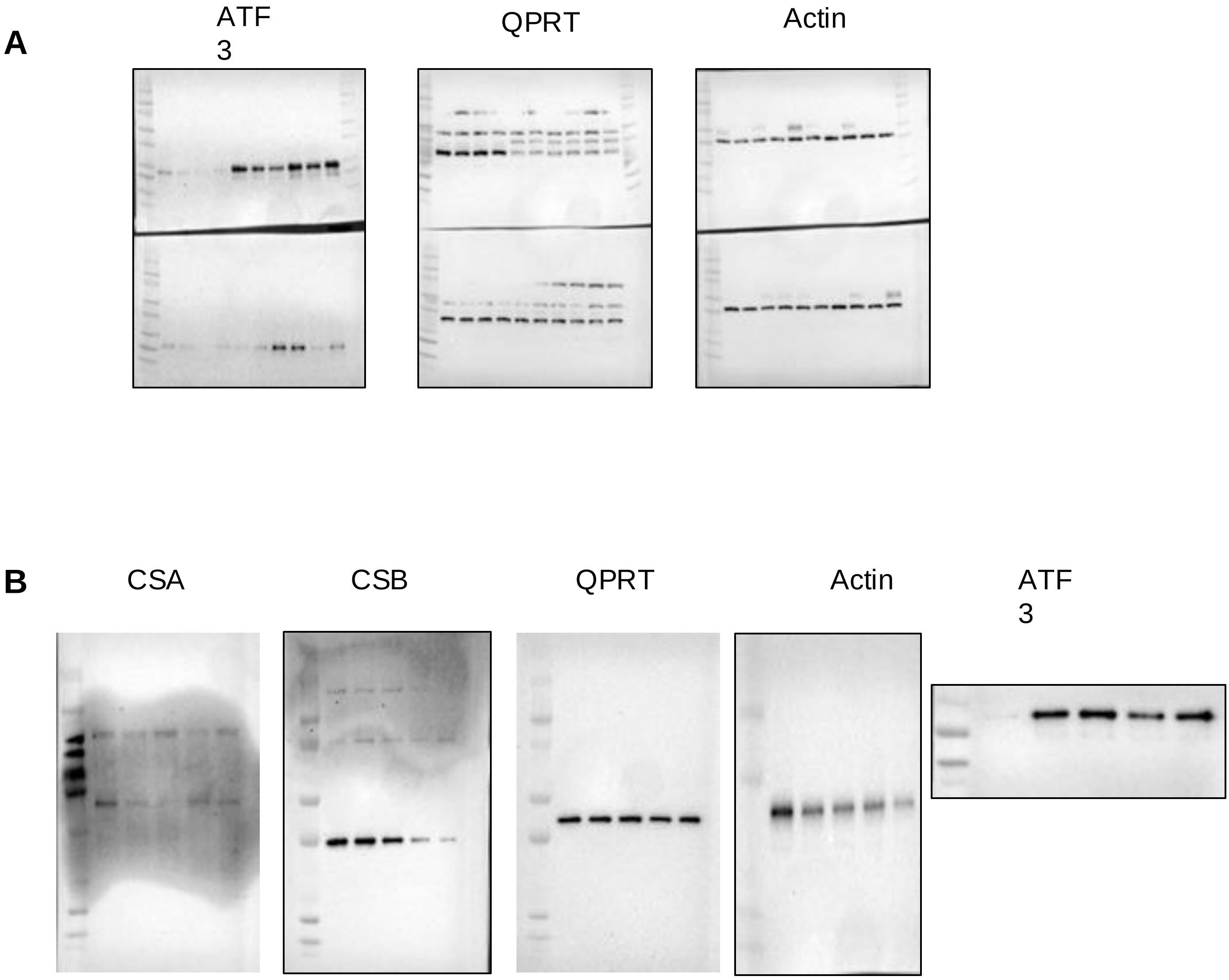

Supplementary Figure 8
Uncropped images of western blot for figure 6 (A) Mouse Kidney tissues (B) HK-2 cells

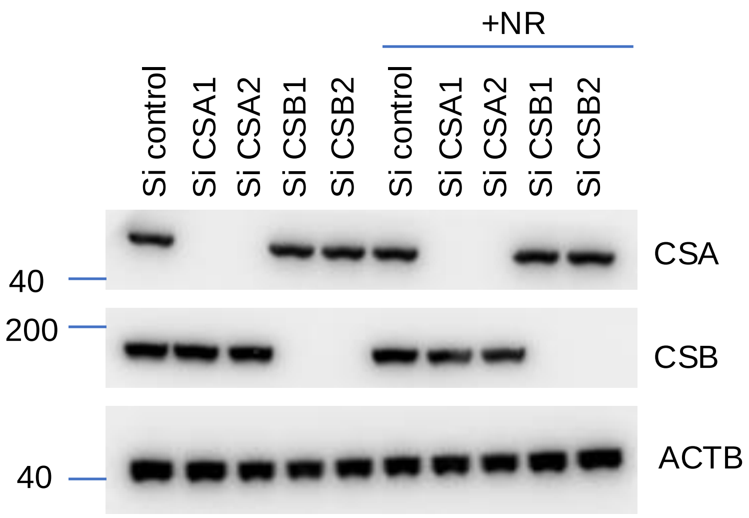

Supplementary Figure 9
HK-2 cells were transfected with siRNA. 72 hours post transfection, cells were supplemented with 1mM NR for 24 hours. Following NR treatment, cells were harvested and analyzed with western blot using indicated antibodies.

Supplementary Table 1

Primers

Primers	Sequences (5'to 3')
**CSA Forward**	GACCTTGAGAACTCCAGCAG
**CSA Reverse**	GTGTCATGAGGATACCACTGT
**CSB Forward**	TCTTGGCAGGTCTGAGCTA
**CSB Reverse**	TGCATCACTGTTGTTGGACA
**mACT Forward**	GATTACTGCTCTGGCTCCTAG
**mACT Reverse**	GACTCATCGTACTCCTGCTTG
**mATF3 Forward**	AGATGTCAGTCACCAAGTCTG
**mATF3 Reverse**	TGTCTTCTCCTTTTTCTTGTTTCG
**mQPRT Forward**	CATGCCACCGCTACGAC
**mQPRT Reverse**	GCTACATTCCACCTCTACCTTC
**mTIMP1 Forward**	AGACAGCCTTCTGCAACTC
**mTIMP1 Reverse**	CAGCCTTGAATCCTTTTAGCATC
**mHAVCR1 Forward**	CTGCTACTGCTCCTTGTGAG
**mHAVCR1 Reverse**	GCGTTCTGCAAAGCTTCAATC
**mLCN2 Forward**	CTACAATGTCACCTCCATCCTG
**mLCN2 Reverse**	CCTGTGCATATTTCCCAGAGT
**hATF3 Forward**	GCTGGAATCAGTCACTGTCAG
**hATF3 Reverse**	CTTATTTCTTTCTCGTCGCCTCT
**hGAPDH Forward**	ACATCGCTCAGACACCATG
**hGAPDH Reverse**	TGTAGTTGAGGTCAATGAAGGG
**ChIP Primer 1 Forward**	TCTCAGGAGTGGTGTGTT
**ChIP Primer 1 Reverse**	AGAAGGTCAATATTTCGTGACA
**ChIP Primer 2 Forward**	GGGAGGGCAGAGCTATTA
**ChIP Primer 2 Reverse**	CCTGGAGCTGTTGTGAAG
**ChIP Primer 3 Forward**	GTGGCTCACACCTGTAAT
**ChIP Primer 3 Reverse**	GACGAGGTTTCTCCATGTT
**ChIP Primer 4 Forward**	CTGGTGATGGTGAAGGATTAAC
**ChIP Primer 4 Reverse**	GCTGCTGCATTGCACTT
**ChIP Primer 5 Forward**	ATCACCCTGGACAACCT
**ChIP Primer 5 Reverse**	AACAGCTTGAGGGAGAAATC
**ChIP Primer 6 Forward**	GCTTGAGCCCAACTCTG
**ChIP Primer 6 Reverse**	CCCATGTGGTTGATCCTTTA
**ChIP Primer 7 Forward**	AGCATTGATTCTTGATCCTCAT
**ChIP Primer 7 Reverse**	TTTCCAGGGATCCAGGTT
**ChIP Primer 8 Forward**	TACCGATGGACCACAGTC
**ChIP Primer 8 Reverse**	GGACAGAGCCACCAGAG
**ChIP Primer 9 Forward**	CCTGTAATCCCAGCACTTTG
**ChIP Primer 9 Reverse**	TTTCGCCATGTTGGTCAG
**ChIP Primer 10 Forward**	CCCGTCTCTACTAAACATACCA
**ChIP Primer 10 Reverse**	AACTCCTGACCTCATGATCC
**hCLU Forward**	GCCTGAAACAGACCTGCAT
**hCLU Reverse**	GTCACCATTCATCCAGAAGTAGA
**hHAVCR1 Forward**	GTCTCTACCTTTGTTCCTCCA
**hHAVCR1 Reverse**	GTTCTCTCCTTATTGCTCCCT
**hLCN2 Forward**	AACTTCATCCGCTTCTCCAA
**hLCN2 Reverse**	TCTCCCAGCTCCCTCAATG
**hNMNAT1 Forward**	TCACCAACATGCACCTCAG
**hNMNAT1 Reverse**	GTAGGCATCACCAACAGGAG
**hNMNAT2 Forward**	GGTGATGCGGTATGAAGAGAT
**hNMNAT2 Reverse**	CACAATCCCAAAGTCACCAAC
**hNMNAT3 Forward**	CTGAGCCCTGCAAATAGCA
**hNMNAT3 Reverse**	GGAGACAAGGCAAGAGTGAA
